# β-catenin represses miR455-3p to stimulate m6A modification of HSF1 mRNA and promote its translation in colorectal cancer

**DOI:** 10.1186/s12943-020-01244-z

**Published:** 2020-08-24

**Authors:** Ping Song, Lifeng Feng, Jiaqiu Li, Dongjun Dai, Liyuan Zhu, Chaoqun Wang, Jingyi Li, Ling Li, Qiyin Zhou, Rongkai Shi, Xian Wang, Hongchuan Jin

**Affiliations:** 1 Department of Medical Oncology, Cancer Institute of Zhejiang University, Sir Run Run Shaw Hospital, School of Medicine, Zhejiang University, Hangzhou, China; 2 Labortary of Cancer Biology, Key Lab of Biotherapy in Zhejiang, Sir Run Run Shaw Hospital, School of Medicine, Zhejiang University, Hangzhou, China; 3 Department of pathology, People’s Hospital of Dongyang, Zhejiang, China

**Keywords:** Colorectal cancer, β-Catenin, HSF1, Translation, miR455-3p, m6A RNA modification

## Abstract

**Background:**

Heat shock transcription factor1 (HSF1) was overexpressed to promote glutaminolysis and activate mTOR in colorectal cancer (CRC). Here, we investigated the mechanism for cancer-specific overexpression of HSF1.

**Methods:**

HSF1 expression was analyzed by chromatin immunoprecipitation, qRT-PCR, immunohistochemistry staining and immunoblotting. HSF1 translation was explored by polysome profiling and nascent protein analysis. Biotin pulldown and m6A RNA immunoprecipitation were applied to investigate RNA/RNA interaction and m6A modification. The relevance of HSF1 to CRC was analyzed in APC^min/+^ and APC^min/+^ HSF1^+/−^mice.

**Results:**

HSF1 expression and activity were reduced after the inhibition of WNT/β-catenin signaling by pyrvinium or β-catenin knockdown, but elevated upon its activation by lithium chloride (LiCl) or β-catenin overexpression. There are much less upregulated genes in HSF1-KO MEF treated with LiCl when compared with LiCl-treated WT MEF. HSF1 protein expression was positively correlated with β-catenin expression in cell lines and primary tissues. After β-catenin depletion, HSF1 mRNA translation was impaired, accompanied by the reduction of its m6A modification and the upregulation of miR455-3p, which can interact with 3′-UTR of HSF1 mRNA to repress its translation. Interestingly, inhibition of miR455-3p rescued β-catenin depletion-induced reduction of HSF1 m6A modification and METTL3 interaction. Both the size and number of tumors were significantly reduced in APC^min/+^ mice when HSF1 was genetically knocked-out or chemically inhibited.

**Conclusions:**

β-catenin suppresses miR455-3p generation to stimulate m6A modification and subsequent translation of HSF1 mRNA. HSF1 is important for β-catenin to promote CRC development. Targeting HSF1 could be a potential strategy for the intervention of β-catenin-driven cancers.

## Introduction

Colorectal cancer (CRC) is the third most common cancer with high mortality rate globally [1]. The accumulation of various genetic and epigenetic changes activates multiple oncogenic signaling critical for the pathogenesis of CRC, such as WNT/β-catenin signaling pathway [2]. Its activation will eventually initiate a transcription-dependent oncogenic process to promote cell cycle progression and apoptosis resistance. While the mechanism for activated WNT/β-catenin signaling to promote CRC development has been well-explored, no therapeutics targeting this pathway has been successfully developed.

In addition to proliferation activation and apoptosis resistance, metabolism reprogramming is one of important hallmarks of cancer cells [3]. For example, cancer cells favor glycolysis instead of oxidative phosphorylation for glucose metabolism even in aerobic conditions, which was well-known as Warburg effect [4, 5]. Pyruvate, the last product of glycolysis, is converted into lactate rather than Acetyl-CoA (acetyl coenzyme A) for TCA (Tricarboxylic acid) cycle or the Krebs cycle. Therefore, targeting enhanced glycolysis has been proposed as novel options in the prevention and treatment of human cancers including CRC [5]. However, as a metabolism hub, TCA cycle is important in both energy production and biosynthesis. Therefore, it needs to be replenished by anaplerotic reactions such as glutaminolysis [6]. Previously, we reported that heat shock transcription factor 1 (HSF1) stimulated glutaminolysis to activate mTOR and promote CRC development by upregulating the expression of glutaminase 1 (GLS1), the critical enzyme in glutaminolysis [7]. HSF1 expression was increased in CRC and had a positive correlation with shorter disease-free survival (DFS). However, the upstream mechanism for HSF1 overexpression in CRC was still unclear.

Gene expression can be controlled by multiple processes including transcription, mRNA degradation, translation and protein degradation. While gene translation and protein degradation have been extensively investigated, more and more studies focused on mRNA translation by exploring the effect of non-coding RNAs such as microRNAs (miRNAs) and new modifications of mRNA including N6-methyladenine (m6A) modification [8, 9]. MiRNAs can form a miRNA-induced silencing complex (miRISC) to posttranscriptionally regulate gene expression by inhibiting cap-dependent initiation and stimulating mRNA deadenylation [10, 11]. On the other hand, as one of the most abundant modifications in mRNA, m6A modification of mRNAs usually promotes translation by recruiting initiation factors such as eIF3 to the 5′ end of the mRNA [12]. While miRNAs and mRNA m6A modifications play a distinct role in mRNA translation, the interplays between them were not clarified.

In this study, we found that activated WNT/β-catenin signaling stimulated HSF1 translation to promote CRC development by repressing HSF1 mRNA-targeting miR455-3p to increase m6A modification of HSF1 mRNA. Therefore, targeting HSF1 translation could be a new strategy for the intervention of CRC and other cancers driven by activated WNT/β-catenin signaling.

## Materials and methods

### Cell, antibodies, and chemicals

Human CRC cell lines SW480, SW620, DLD1, RKO were obtained from the American Type Culture Collection (ATCC). All cells were routinely cultured in RPMI 1640 (Invitrogen; 11875–093) or DMEM (Invitrogen; 11965–092) supplemented with 10% fetal bovine serum. All cells were incubated at 37 °C with 5% CO2 and 95% humidity. The following antibodies were used for western blotting and IHC: HSF1 (12972S, Cell Signaling Technology, CST; ab52757, Abcam); β-catenin (8480S, CST); β-actin (4970 L, CST); FLAG (F1804–1, Sigma); cyclinD1 (ab134175, Abcam); Cleaved PARP1 (9541-s, CST); METTL3 (a8370, Abclonal); GLS1 (ap8809b, Abgent). Pyrvinium (P0027), LiCl (793620), cycloheximide (R750107), chloroquine (C6628), MG132 (474790) and PD150606 (D5946) were purchased from Sigma-Aldrich.

### SiRNA, miRNA mimics/inhibitors transfection

Small interfering RNA (siRNA) targeting β-catenin, METTL3 and microRNAs were synthesized by Genepharma (Shanghai, China) and RiboBio (Guangzhou, China). The sequence of these siRNAs and miRNAs were listed in Additional file 1: Table S1. SiRNAs and miRNA mimics/inhibitors were transfected into cells seeded overnight by lipo2000 (Invitrogen, USA) or Lipofectamine RNAiMax transfection reagent (Invitrogen, USA), according to the manufacturer’s instructions.

### Luciferase activity assay

The plasmid of β-catenin reporter was gifted from Prof. Ximei Wu (Zhejiang University). For HSF1 activity assay, a fragment containing 3 X HSE were synthesized and inserted into the PGL3-basic vectors (Promega Corporation, USA). The plasmid was co-transfected with pRL renilla and β-catenin siRNA by using lipo2000 (Invitrogen, USA) or treatment with pyrvinium by X-treme GENE HP DNA Transfection Reagent (Roche, USA). 3′-UTR segment of the HSF1 was cloned by PCR and inserted into the vector pMIR-REPORTER (Promega). The mutation of miR455 binding sites in HSF1 3′-UTR was generated by Quick Site-Directed Mutagenesis (600674–51, Stratagene, USA). The resultant plasmids were co-transfected with pRL renilla and miR455 mimics by using lipo2000 (Invitrogen, USA). 48 h post-transfection, the luciferase activity was measured by the Dual-GLO Luciferase Assay System (Promega Corporation, USA).

### Chromatin immunoprecipitation (ChIP)

ChIP analysis was conducted with the SimpleChIP™ Enzymatic Chromatin IP Kit (CST, USA). Antibodies used were anti-HSF1 (12972S, CST), TCF7L2(C48H11, CST) and FLAG (F1804–1, Sigma). The primers used for the PCR analysis of precipitated DNA were shown in Additional file 1: Table S2. For FLAG-ChIP assay, the flag-β-catenin vector was transiently overexpressed by transfection. After 48 h, the enrichment of flag-β-catenin on COL27A1 promoter was measured by the ChIP Kit.

### Immunoblotting and immunohistochemistry

Immunoblotting and immunohistochemistry (IHC) assays were performed as previously reported [7]. For Immunoblotting, total proteins were extracted with RIPA buffer supplemented with protease inhibitors (Roche, USA). After heating the protein sample to 95–100 °C for 20 min, cell lysates were transferred to polyvinylidene fluoride (PVDF) membranes. After the membranes were blocked in 5% milk, primary antibody with gentle agitation overnight at 4 °C.

For IHC assay was performed in a tissue array containing 80 cases of colonic tissues. Primary antibodies used were listed above. The degree of immunostaining was assessed by 2 independent pathologists and evaluated by assigning a score of 0–3. Scores were defined as follows: 0, no staining; 1, faint staining; 2, moderate staining and 3, strong staining. Final scores of 0 and 1 were regarded as low expression, whereas scores of 2 and 3 considered as high expression.

### Apoptosis detection

Cell apoptosis was measured by flow cytometry analysis and western blotting. For flow cytometry analysis of apoptosis, cells were harvested and re-suspended in 100 μl 1 x binding buffer. 5 μl fluorescein isothiocyanate (FITC) annexin V and propidium iodide (PI) (556,547; BD Biosciences, USA) were added to the cell suspension and then incubated for 15 min at room temperature. After that, the samples were attenuated with 400ul 1 x binding buffer and analyzed by ACS Calibur flow cytometer.

### Puromycin-labelling

To detect the change of nascent HSF1 synthesis, 2 X 10^6^ cells were plated in 10 cm dish. After the given treatment, cells were incubated with 1:1000 Biotin-dC-puromycin (NU-925-BIO-S, Jena Bioscience) for 24 h. Cells were lysed with 1% NP40 buffer (20 mM Tris-HCl, PH 7.4, 150 mM NaCl, 1% NP40, 10% glycerol) containing 1X protease inhibitor cocktail. After adequate centrifugation, the supernatant was incubated with 80ul streptavidin sepharose beads (GE17–5113-01, Sigma) by rotating at 4 °C for 6 h to overnight. The mixture was washed by 1% NP40 buffer for 5 times and subjected to western blotting using HSF1 antibody.

### Polysome profiling

Polysome profiling separates translating or non-translating mRNAs on a sucrose gradient according to the number of bound ribosomes as previously described [13]. In brief, cells were grown to ~ 80% confluence. Before collection, cells were incubated with 100 μg/ml of cycloheximide for 15 min. Then cells are lysed by polysome buffer [200 mmol/L KCl, 15 mmol/L MgCl2, 1% Triton X100, 100 μg/mL cycloheximide, 20 mmol/L heparin, and 100 U/mL RNase Inhibitor (Takara), 1X cocktail] for 15 min on ice, lysates were centrifuged (14,000 rpm for 15 min), and the supernatant was layered onto a 5 to 50% sucrose gradient. Gradients were then centrifuged at 38,000 rpm for 130 min at 4 °C and polysome-bound fractions were collected using an ISCO Density Gradient Fractionation System (ISCO, Lincoln, NE) with continuous monitoring based on A260nm wavelength. The RNA in each fraction was extracted using Trizol reagent (Invitrogen) and analyzed by real-time RT-PCR.

### Biotin pull down assay

Biotin pull down assay was performed as described previously [14]. Cells were transfected with biotinylated miR455-3p probes for 48 h and resuspended using lysis buffer (20 mM Tris, pH 7.5, 200 mM NaCl, 2.5 mM MgCl2, 60 U/mL, SUPERase-In, 1 mM DTT, 0.05% Igepal, protease inhibitors). Lysates were incubated with prepared streptavidin beads (GE Healthcare). Yeast tRNA (Sigma) was used for blocking lysates at 4 °C for 3 h. Then washed 5 times with binding and wash buffer (5 mM Tris-HCl, pH 7.5, 0.5 mM EDTA, 1 M NaCl). Finally, the bound RNAs were extracted and purified for qPCR.

### RNA immunoprecipitation (RIP) assay

RIP assay was performed by Magna RIPTM RNA-Binding Protein Immunoprecipitation Kit (Millipore, No.17–700). Briefly, 2 × 10^7^ cells were lysed in 100 μl RIP lysis buffer and immunoprecipitated with antibodies of interest and protein G magnetic beads at 4 °C overnight, followed by six times of washes in Washing Buffer and protein digestion at 55 °C. Total RNA was isolated and subjected to RT-PCR analysis. Following antibodies were used for RIP: N6-methyladenosine (202,003, synaptic); METTL3 (a8370, Abclonal); IgG (2,912,787, Millipore).

### RNA-sequencing

2X10^6^ WT MEF and HSF1 KO MEF were plated and cultured overnight. Following day, cells were treated with 20 mM LiCl for 48 h. Cells were collected with Trizol reagent. The total RNA was processed by NEBNext® Poly(A) mRNA Magnetic Isolation Module to enrich mRNA, and the product RNA was used for construction Library, via KAPA Stranded RNA-Seq Library Prep Kit (Illumina). Sequencing libraries, denatured by 0.1 M NaOH to generate single-stranded DNA, as amplified in situ Illumina cBot (TruSeq SR Cluster Kit v3-cBot-HS (# GD-401-3001, Illumina)). The ends of the generated fragments were used to run 150 Cycles by the Illumina HiSeq 4000 Sequencer. All the experimental steps after the RNA extraction were conducted in Kangcheng Biotechnology Co., Ltd. (Aksomics), Shanghai, China. RNA-sequencing was performed three times.

### Animal experiments

Animal care and experiments were conducted in compliance with Institutional Animal Care and Use Committee and NIH guidelines. The C57BL/6 J mice and Apc^min/+^ mice were purchased from Model Animal Research Center of Nanjing University (MARC, Nanjing, China). HSF1 KO mice reported previously were used to generate Apc^min/+^ mice HSF1^+/−^ [7]. Subsequently, 4 groups of mice, wild type, Apc^min/+^, Apc^min/+^ HSF1^+/−^ and Apc^min/+^ treated with KNK437 as previously reported [7], were fed with high-fat diet (45 Kcal% Fat) for 3 months. The intestine was dissected, flushed with PBS and cut open longitudinally along the main axis. The number of tumors was counted and the sizes of tumors were measured.

### Statistics

All data were expressed as mean ± SD. Unless specified, the Student’s t-test was performed for statistical significance analysis. *P* value < 0.05 was considered as statistically significant.

## Results

### β-Catenin activates HSF1 in CRC

**Fig. 1:**
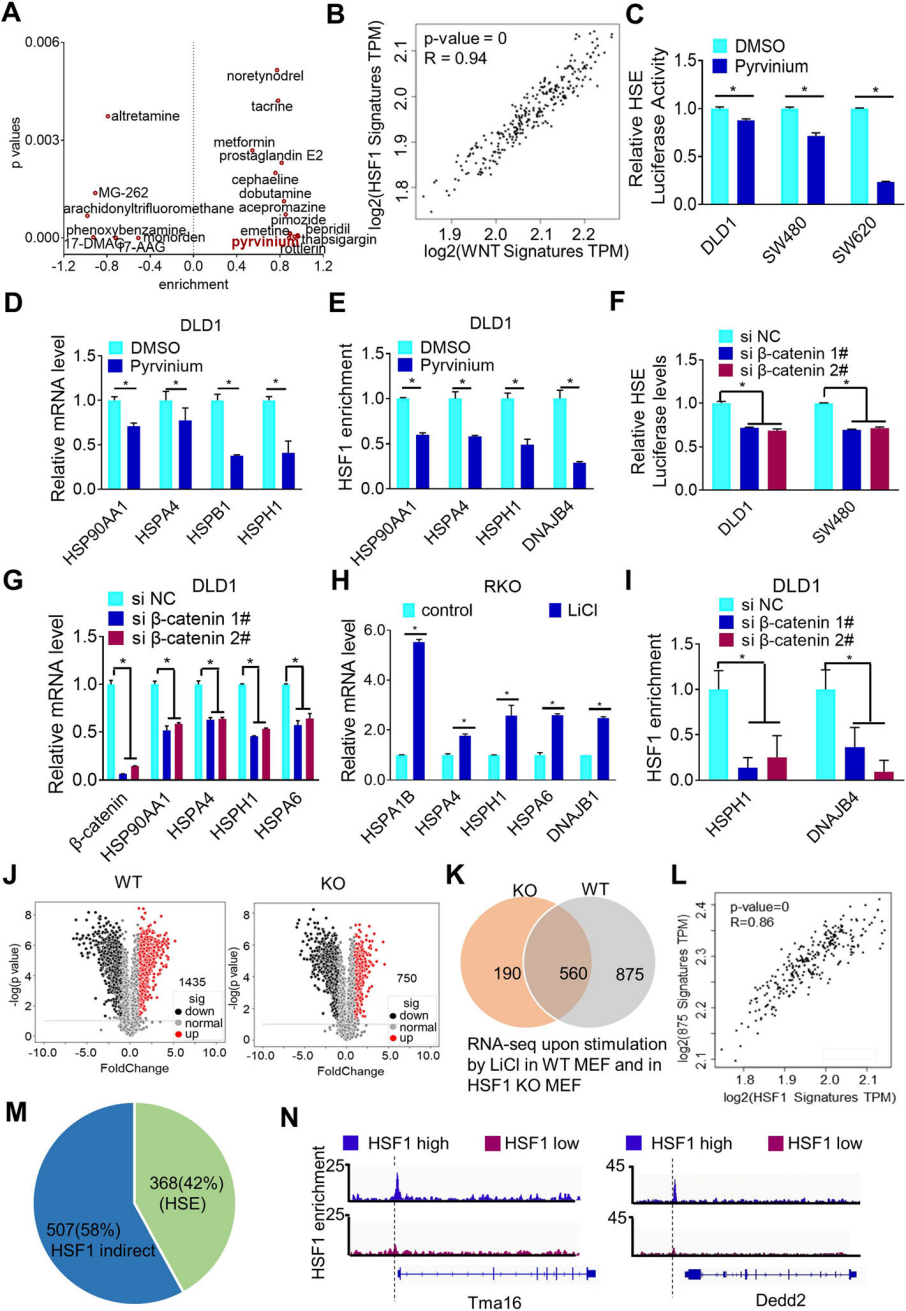
WNT/β-catenin signaling activates HSF1. **a** Chemicals influencing gene expression in a similar manner to HSF1 inhibition were screened by connective map analysis. **b** The correlation of WNT/β-catenin signaling signature and HSF1 signature was detected by GEPIA. **c** The effect of Pyrvinium on HSE-driven promoter activity was explored by luciferase reporter assay. **d** The effects of Pyrvinium on the targets of HSF1 were analyzed by RT-PCR. **e** Binding of HSF1 to the promoters of HSF1 targets in CRC cells treated with or without Pyrvinium was determined by ChIP. **f** The luciferase assays of HSE before and after β-catenin knockdown were shown as in C. **g** and **h** The mRNA levels of HSF1 targets with β-catenin knockdown or LiCl treatment were analyzed by RT-PCR. **i** Binding of HSF1 to its targets promoter in CRC cells before and after β-catenin knockdown was analyzed by ChIP. **j** Volcano plot displays differentially regulated genes in dHSF1 compared to WT parental cells with LiCl. Red dots indicate significantly regulated genes based on adjusted *p*-value and log-fold change (logFC) (*p* < 0.01, log2FC > 2). **k** Differential gene expression analysis in WT and HSF1+/− MEF treated with LiCl were performed by RNA-seq. Numbers of upregulated genes in two cells were shown in Venn graph. **l** The correlation of 875 putative HSF1-dependent genes from K with reported HSF1 signature was detected by GEPIA. **m** Numbers of 875 putative HSF1-dependent genes with or without HSE in their promoters were summarized. **n** Representative HSF1 ChIP-seq tracks (NCBI GEO: GSE57398) for 368 HSE-containing genes are shown. Asterisks (*) indicate *p* < 0.05

In an effort to explore potential regulations of HSF1, we screened chemicals generating a gene expression pattern similar to HSF1 depletion by connective map (http://portals.broadinstitute.org/cmap/) [7, 15, 16]. Interestingly, a recently reported inhibitor of WNT/β-catenin signaling, pyrvinium, had a similar effect on genome-wide gene expression as HSF1 depletion [17] (Fig. 1a and Additional file 2, Fig.S1A-B). Moreover, the expression signature related to WNT/β-catenin signaling [18] was positively correlated with the HSF1 signature [15] (Fig. 1b), indicating a potential connection of HSF1 with WNT/β-catenin signaling. Indeed, pyrvinium attenuated the activity of a luciferase reporter driven by HSF1 binding sites (HSE, heat shock response elements) [19, 20] (Fig. 1c) and reduced the expression of well-known transcriptional targets of HSF1 such as HSP90AA1, HSPA4, HSPB1, and HSPH1 (Fig. 1d and Additional file 2, Fig.S1C). Chromatin immunoprecipitation (ChIP) assay further confirmed the reduced interaction of HSF1 with its transcriptional targets (Fig. 1e and Additional file 2, Fig.S1D). In consistence with pyrvinium, knock-down of β-catenin by siRNA also decreased HSF1 activity (Fig. 1f), reduced the expression of HSF1 targets (Fig. 1g and Additional file 2, Fig.S1E) and attenuated the interaction of HSF1 with its targets (Fig. 1i and Additional file 2, Fig.S1F). Furthermore, HSF1 targets were upregulated by the potent GSK3β inhibitor LiCl in colorectal cancer cell line RKO, which had a low level of β-catenin expression (Fig. 1h).

To explore the biological relevance of HSF1 activation to β-catenin signaling, we profiled gene expression of wild type mouse embryonic fibroblasts (WT MEF) and HSF1 knock-out MEF (HSF1 KO MEF) before and after LiCl treatment (NCBI GEO: GSE151119). While only 750 genes were upregulated in LiCl treated-HSF1 KO MEF, there were 1435 genes significantly upregulated in WT MEF after LiCl treatment (Fig. 1j). Among them, 875 genes displayed a dependence on HSF1 since their expression levels failed to be upregulated by LiCl treatment once HSF1 was depleted (Fig. 1k). In fact, their expression levels had a high correlation with the expression of a previously reported HSF1 signature (Fig. 1l). Furthermore, 368 (42%) genes had a HSE (Heat shock response element, HSE) within their promoter regions (Fig. 1m and Additional file 3), meaning that they are most likely bona fide targets of HSF1. Indeed, some of them such as Tma16, Dedd2, Hspa9 and Kif21a have been confirmed as the target of HSF1 by ChIP-seq (NCBI GEO: GSE57398) (Fig. 1n and Additional file 2, Fig.S1G). Taken together, these results indicated that β-catenin can positively regulate HSF1.

### β-Catenin stimulates HSF1 protein translation

**Fig. 2:**
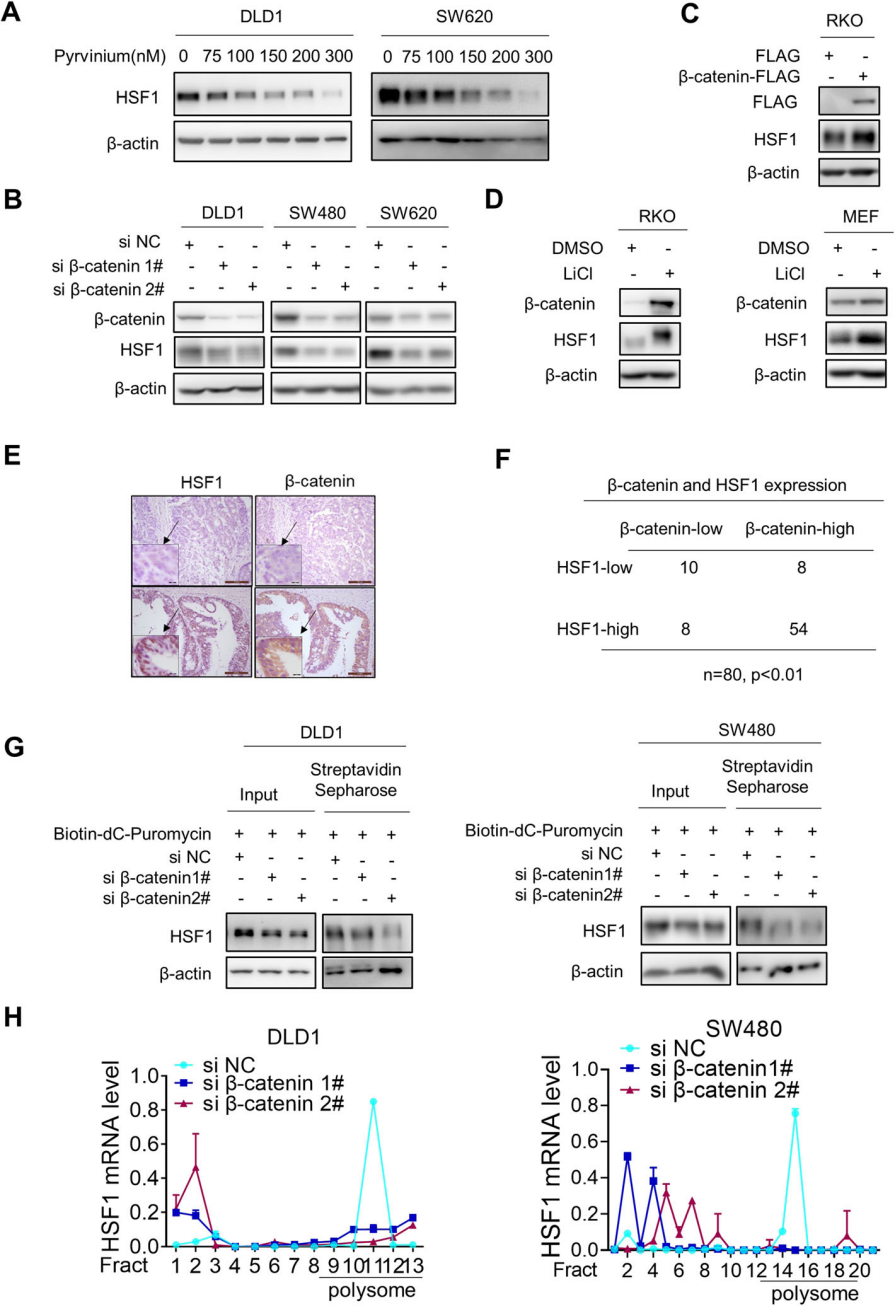
β-catenin stimulates HSF1 protein translation. **a** The effect of Pyrvinium on the protein expression of HSF1 was explored by western blotting. **b** The effect of β-catenin knockdown on HSF1 protein level was analyzed by western blotting. **c** The protein level of HSF1 before and after β-catenin overexpression was analyzed by western blotting. **d** The effect of LiCl on HSF1 protein level in RKO and MEF was analyzed by western blotting. **e** The expression of β-catenin and HSF1 in colorectal tissue was analyzed by immunohistochemistry staining. **f** The correlation between β-catenin expression and HSF1 expression in colorectal tissue was analyzed by chi-square test (*p < 0.01). **g** The effect of β-catenin depletion on HSF1 with puromycin labeling was determined by western blotting. **h** Amount of HSF1 mRNA in various polysome fractions was analyzed by RT-PCR(*p < 0.05)

To delineate how β-catenin regulates HSF1, we quantitated protein levels of HSF1 before and after inhibiting β-catenin. Both pyrvinium and β-catenin depletion reduced the protein level of HSF1 (Fig. 2a and b). In contrast, overexpression of exogenous β-catenin increased HSF1 protein level (Fig. 2c). Furthermore, HSF1 protein level was increased after activating WNT/β-catenin signaling by LiCl treatment in both RKO and MEF cells (Fig. 2d). In addition, HSF1 expression correlates well with β-catenin expression in primary tissues (Fig. 2e and f, *p* < 0.01, Chi-Square Test). All of these data indicated that β-catenin upregulates HSF1 protein expression.

However, there were no apparent alterations in HSF1 mRNA level after β-catenin knockdown or pyrvinium treatment (Additional file 2, Fig.S2A and S2B). Meanwhile, the half-life of HSF1 protein was also not changed before and after β-catenin knockdown (Additional file 2, Fig.S2C). Inhibitors of proteasome, autophagy and calpains all failed to reverse HSF1 protein downregulation induced by β-catenin knockdown (Additional file 2, Fig.S2D). All of these results implied that β-catenin affects HSF1 protein expression most-likely via translation regulation. Therefore, puromycin labeling assay was employed to monitor the synthesis of nascent HSF1 protein [21]. As expected, the puromycin labeling of HSF1 was reduced by β-catenin depletion (Fig. 2g). To further confirm it, mono/polysome fractions from cytoplasmic extracts of CRC cells before and after β-catenin depletion were collected by sucrose gradient centrifugation. The subsequent RT-PCR analysis revealed that β-catenin depletion considerably reduced the presence of HSF1 mRNA in the polysome fraction but increased in non-translating ribosome fractions (Fig. 2h). In summary, β-catenin upregulates HSF1 expression by stimulating HSF1 protein translation.

### HSF1 protein translation is regulated by miR455-3p

**Fig. 3:**
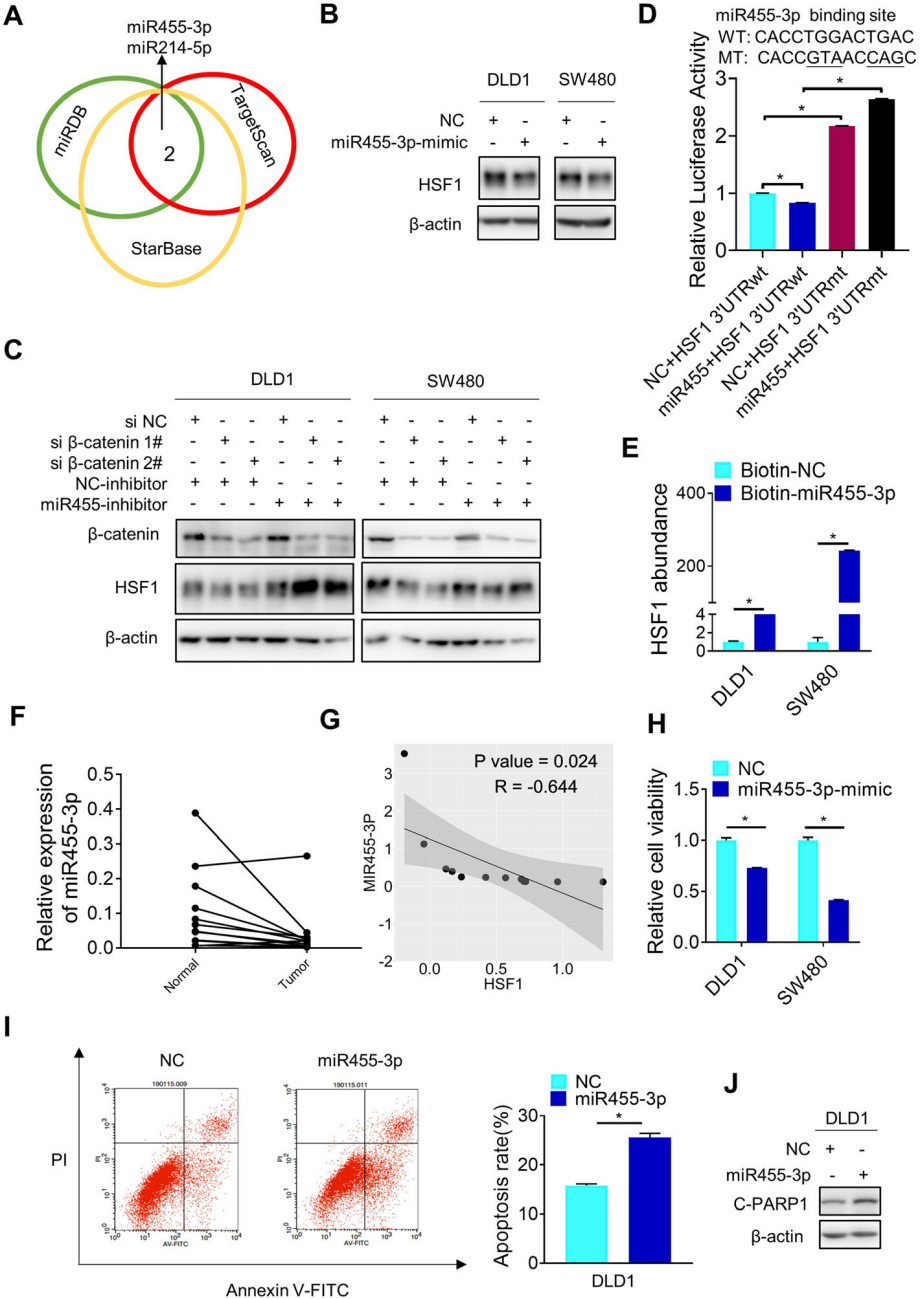
HSF1 protein translation is regulated by miR455-3p. **a** Overlap of HSF1-targeting microRNAs predicted by TargetScan, miRDB and StarBase. **b** The effect of miR455-3p on HSF1 protein was analyzed by western blotting. **c** The effect of miR455-3p inhibitor on β-catenin knockdown-induced HSF1 downregulation was determined by western blotting. **d** Luciferase activity assay was used to analyze the effect of miR455-3p on the activity of 3′-UTR with or without miR455-3p binding sites (*p < 0.05). **e** The binding between biotin-miR455-3p and HSF1 mRNA was determined by biotin pull down assay (*p < 0.05). **f** Expression of miR455-3p in 12 pairs of fresh CRC tissues and adjacent non-tumor tissues was analyzed by qPCR. **g** The correlation of HSF1 protein and miR455-3p in 12 pairs of fresh CRC tissues and adjacent non-tumor tissues was analyzed. **h** The effect of miR455-3p on viability of CRC cells was explored by MTS assay. **i** The effect of miR455-3p on apoptosis of CRC cells was analyzed using flow cytometry after PI and annexin V-FITC double staining. **j** Apoptosis of CRC cells treated with or without miR455-3p was determined by western blotting

As microRNAs (miRNAs) play an important role in regulating the efficiency of protein translation, we wondered whether HSF1 protein translation was regulated by microRNAs. Based on bioinformatics screening by TargetScan, miRDB and StarBase, some microRNAs including miR455-3p, miR214-5p, miR431-5p, miR184, miR490-3p, and miR375 were proposed to target 3′-UTR of HSF1 mRNA (Fig. 3a). After functional validation by western blotting, miR455-3p and miR214-5p, but not other microRNAs, were capable to suppress the expression of HSF1 protein in CRC cells (Fig. 3b and Additional file 2, Fig.S3A). However, miR214-5p but not miR455-3p also reduced HSF1 mRNA level (Additional file 2, Fig.S3B). What’s more, an inhibitor of miR455-3p rather than miR214-5p rescued the downregulation of HSF1 protein by β-catenin knockdown (Fig. 3c and Additional file 2, Fig.S3C), indicating that miR455-3p might be relevant to β-catenin-involved regulation of HSF1 protein translation. Indeed, miR455-3p inhibited the activity of luciferase driven by wild type HSF1 mRNA 3′-UTR but not its mutant unable to bind miR455-3p (Fig. 3d). The interaction of miR455-3p with HSF1 mRNA was further confirmed by biotin pull down assay (Fig. 3e). Based on the analysis of TCGA data (http://mirtv.ibms.sinica.edu.tw/), the expression of miR455-3p is lower in colon adenocarcinoma than in normal tissues (Additional file 2, Fig.S3D). Similarly, qPCR analysis revealed lower levels of miR455-3p in CRC tissues than in adjacent non-tumor tissues (Fig. 3f). Additionally, we had confirmed high expression of HSF1 in the same cohort of human CRC tissues previously [7]. Indeed, miR455-3p expression was negatively correlated with the expression of HSF1 (Fig. 3g). On the other hand, miR455-3p, similar to HSF1 inhibition as we reported recently [7], reduced the expression of HSF1 targets, induced the viability inhibition and apoptosis activation of colorectal cancer cells (Fig. 3h-j and Additional file 2, Fig.S3E-G). The seed sequence of microRNAs was important for targeting mRNA by base-pairing [22]. Indeed, the seed sequence mutant of miR455-3p could not downregulate the protein level of HSF1 (Additional file 2, Fig.S4A), confirming the importance of miR455-3p to target HSF1 protein expression. In a word, miR455-3p targets HSF1 mRNA 3′-UTR to inhibit its translation.

### m6A modification of HSF1 mRNA stimulates its protein translation

**Fig. 4:**
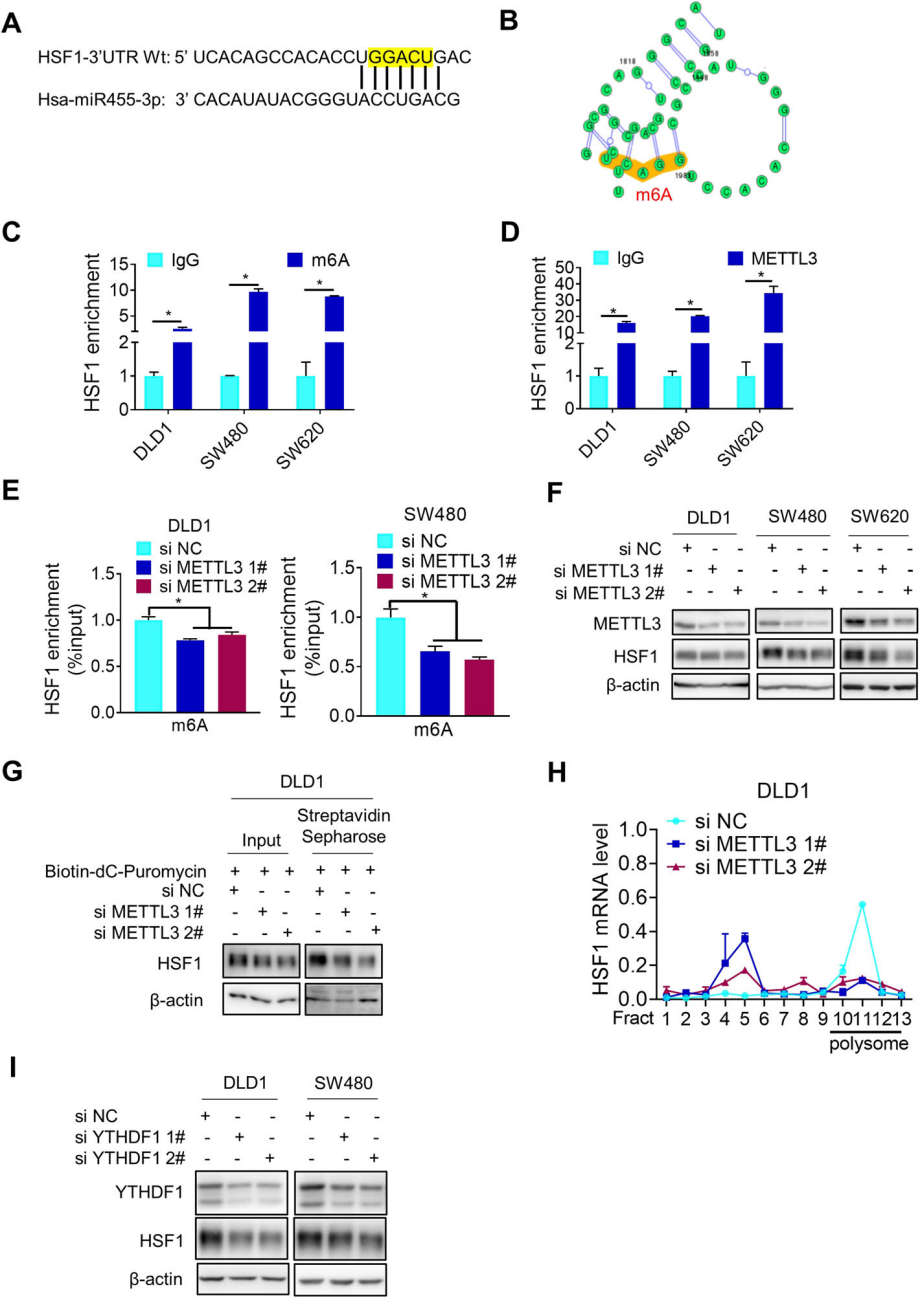
m6A modification of HSF1 mRNA stimulates its protein translation. **a** The sites of HSF1 3′-UTR binding to the seed sequence of miR455-3p was consistent with m6A RNA modification elements “DRACH”. **b** Bioinformatic prediction of m6A modification in 3′-UTR of HSF1 mRNA. **c** m6A modification of HSF1 mRNA was analyzed by meRIP (*p < 0.05). **d** Binding of METTL3 to HSF1 mRNA was detected by RIP (*p < 0.05). **e** m6A modification of HSF1 mRNA with or without METTL3 depletion was analyzed by meRIP (*p < 0.05). **f** The protein level of HSF1 before and after METTL3 depletion was detected by western blotting. **g** The effect of METTL3 knockdown on HSF1 synthesis was determined by western blotting after puromycin labeling. **h** Amount of HSF1 mRNA in various polysome fractions was analyzed by RT-PCR(*p < 0.05). **i** The effect of YTHDF1 knockdown on HSF1 protein level was analyzed by western blotting

In addition to microRNA, mRNA modifications such as N6-methyladenosine (m6A) play important roles in the regulation of HSF1 translation. Interestingly, we noticed that the matching sites of miR455-3p seed sequence in HSF1 mRNA 3′-UTR contains a typical motif of m6A modification (Fig. 4a), which was supported by bioinformatic analysis (http://www.cuilab.cn/sramp) (Fig. 4b and Additional file 2, Fig.S4B). Moreover, we have done the MeRIP sequencing in SW620 and found that the 3′-UTR region of HSF1 has one m6A modification site. Intriguingly, this sequence is completely complementary to the seed sequence of miR455-3p (Additional file 2, Fig.S4C). PCR analysis after meRIP (m6A RNA immunoprecipitation) further confirmed m6A modification of HSF1 mRNA (Fig. 4c). What’s more, the activity of luciferase driven by the mutant HSF1 mRNA 3′-UTR, which was unable to bind miR455-3p but retains the m6A modification site sequence DRACH (D = A, G or U; R = A or G; H = A, U or C) [23–25], was higher than the activity of luciferase driven by wild type HSF1 mRNA 3′-UTR (Fig. 3d), indicating the importance of m6A modification to HSF1 expression. As the main component of the methyltransferase “writer” complex [26, 27], METTL3 was also bound to HSF1 mRNA (Fig. 4d). Once its expression was depleted, m6A modification of HSF1 mRNA was decreased (Fig. 4e). In consistence with its potential roles in promoting protein translation, such a reduction of HSF1 mRNA m6A modification reduced HSF1 protein expression (Fig. 4f) and nascent HSF1 protein synthesis (Fig. 4g). Furthermore, METTL3 depletion considerably reduced the presence of HSF1 mRNA in the polysome fraction but increased in non-translating ribosome fractions (Fig. 4h), while HSF1 mRNA or the stability of HSF1 protein were not changed (Additional file 2, Fig.S4D and S4E). Moreover, HSF1 protein was decreased with the knockdown of YTHDF1, which was the reader protein of HSF1 m6A modification (Fig. 4i). To sum up, m6A modification of HSF1 mRNA was relevant to stimulate its translation.

### β-Catenin suppresses miR455-3p to increase HSF1 mRNA m6A modification

**Fig. 5:**
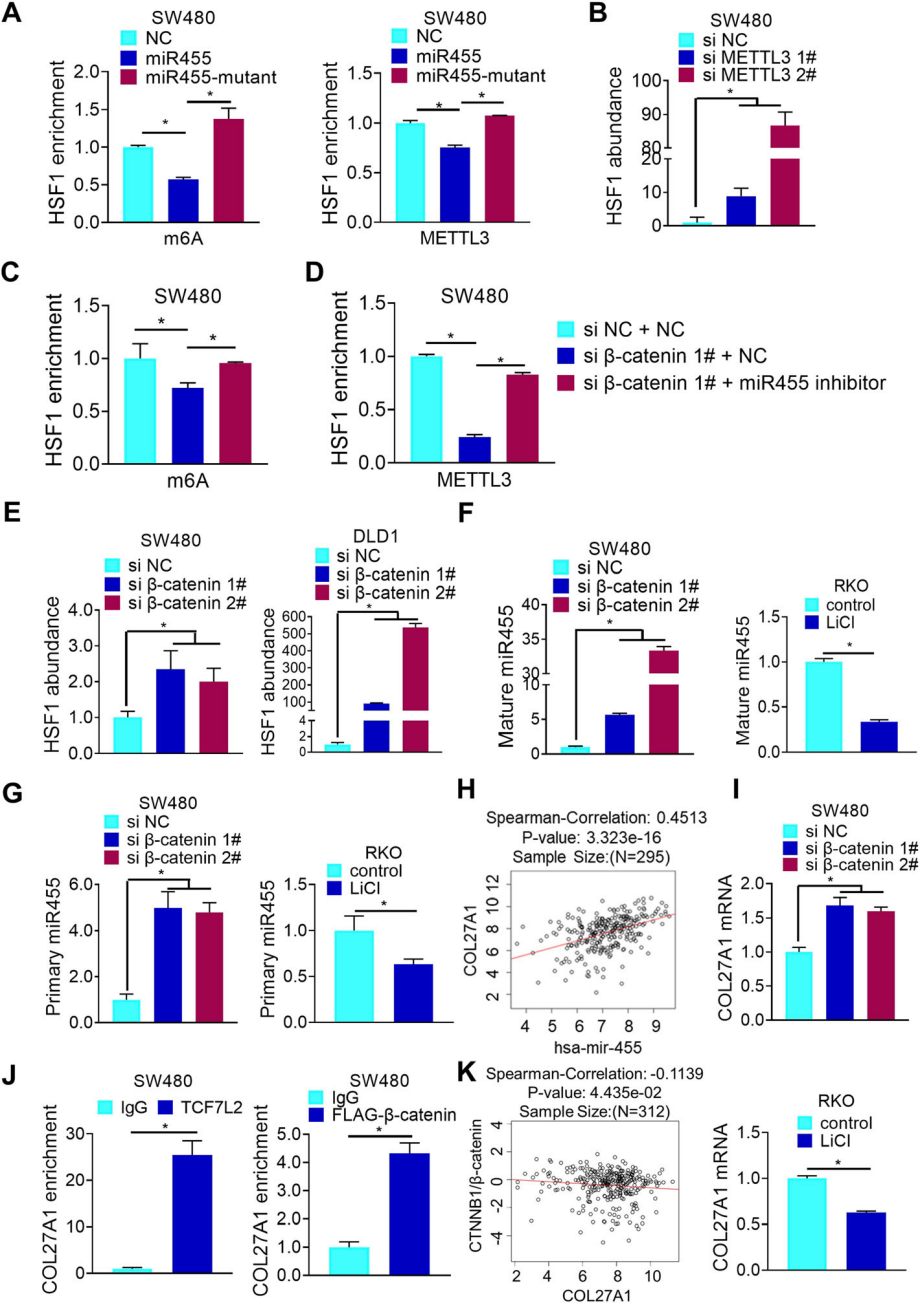
β-catenin suppresses miR455-3p to increase HSF1 mRNA m6A modification. **a** m6A modification and METTL3 interaction of HSF1 mRNA with WT or mutant of miR455-3p were analyzed by RIP. **b** The interaction between biotin-miR455-3p and HSF1 mRNA with or without METTL3 depletion was analyzed by biotin pull down. **c** The effect of miR455-3p inhibitor and/or β-catenin knockdown on m6A modification of HSF1 mRNA was analyzed by meRIP. **d** The effect of miR455-3p inhibitor and/or β-catenin knockdown on the interaction of METTL3 with HSF1 mRNA was analyzed by RIP. **e** The effect of β-catenin knockdown on interaction of miR455-3p and HSF1 mRNA was analyzed by biotin pull down. **f** and **g** The levels of mature (**f**) and primary (**g**) miR455-3p with β-catenin knockdown or LiCl treatment were determined by RT-PCR. **h** The correlation of COL27A1 and miR455 was analyzed in linkedomics (http://linkedomics.org/). **i** The effect of β-catenin depletion or LiCl on mRNA level of COL27A1 was analyzed by RT-PCR. **j** The interaction of β-catenin/TCF7L2 and HSF1 promoter was determined by ChIP. **k** The correlation of β-catenin protein expression with the RNA level of COL27A1 was detected by linkedomics (http://linkedomics.org/)

Next, we explored the interplay between miR455-3p and m6A modification of HSF1 mRNA. Both HSF1 mRNA m6A modification and its binding to METTL3 were decreased by the overexpression of wild type miR455-3p but not its mutant unable to bind to HSF1 mRNA 3′-UTR (Fig. 5a and Additional file 2, Fig.S5A). Interestingly, METTL3 depletion not only reduced m6A modification of HSF1 mRNA (Fig. 4e), but also enhanced the interaction of miR455-3p with HSF1 mRNA (Fig. 5b and Additional file 2, Fig.S5B), while the expression of mature and primary miR455-3p was not upregulated (Additional file 2, Fig.S5C-D). These results indicated that miR455-3p may compete with METTL3 for the m6A modification of HSF1 mRNA, thus inhibiting HSF1 protein translation. Furthermore, the binding of miR455-3p to HSF1 mRNA was not changed by YTHDF1 deletion (Additional file 2, Fig.S5E), indicating that the translation repression of HSF1 mRNA was more likely to be mediated directly by the reduced m6A modification of HSF1.

Indeed, miR455-3p inhibitor rescued β-catenin depletion-induced decrease of HSF1 mRNA m6A modification (Fig. 5c and Additional file 2, Fig.S5F). Meanwhile, the interaction of METTL3 with HSF1 mRNA was abrogated by depleting β-catenin (Fig. 5d and Additional file 2, Fig.S5G), accompanied by the increased interaction of miR455-3p to HSF1 mRNA (Fig. 5e), and upregulation of mature (Fig. 5f and Additional file 2, Fig.S5H), precursor (Additional file 2, Fig.S5I) and primary miR455-3p (Fig. 5g and Additional file 2, Fig.S5J). In contrast, when β-catenin was upregulated by overexpression or LiCl treatment, both mature miR455-3p (Fig. 5f and Additional file 2, Fig.S5H) and primary miR455-3p (Fig. 5g and Additional file 2, Fig.S5J) were downregulated.

Primary miR455-3p was derived from a pre-miRNA hairpin encoded in intron 10 of the collagen gene COL27A1 [28](Additional file 2, Fig.S5K). Actually, COL27A1 expression was significantly correlated with the expression of miR455 (http://linkedomics.org/) (Fig. 5h). Consistent with this, we observed COL27A1 mRNA levels were increased upon β-catenin depletion while decreased after LiCl treatment (Fig. 5i and Additional file 2, Fig.S5L). Moreover, β-catenin/TCF7L2 complex could interact with the promoter of COL27A1, while the pair of primers (Negative-chip-primer) at a position far away from the promoter region could not enrich COL27A1 (Fig. 5j and Additional file 2, Fig.S5M). Meanwhile, the protein expression of β-catenin was negatively correlated with RNA level of COL27A1 (http://linkedomics.org/) (Fig. 5k). These results indicated that the transcription of COL27A1 was inhibited by WNT/β-catenin signaling, leading to decreased biogenesis of miR455-3p. Therefore, β-catenin facilitates the shift from miR455-3p binding to m6A modification in HSF1 mRNA by suppressing miR455-3p expression, eventually promoting HSF1 protein translation.

### Both genetic and chemical inhibition of HSF1 attenuate colorectal carcinogenesis in mice

**Fig. 6:**
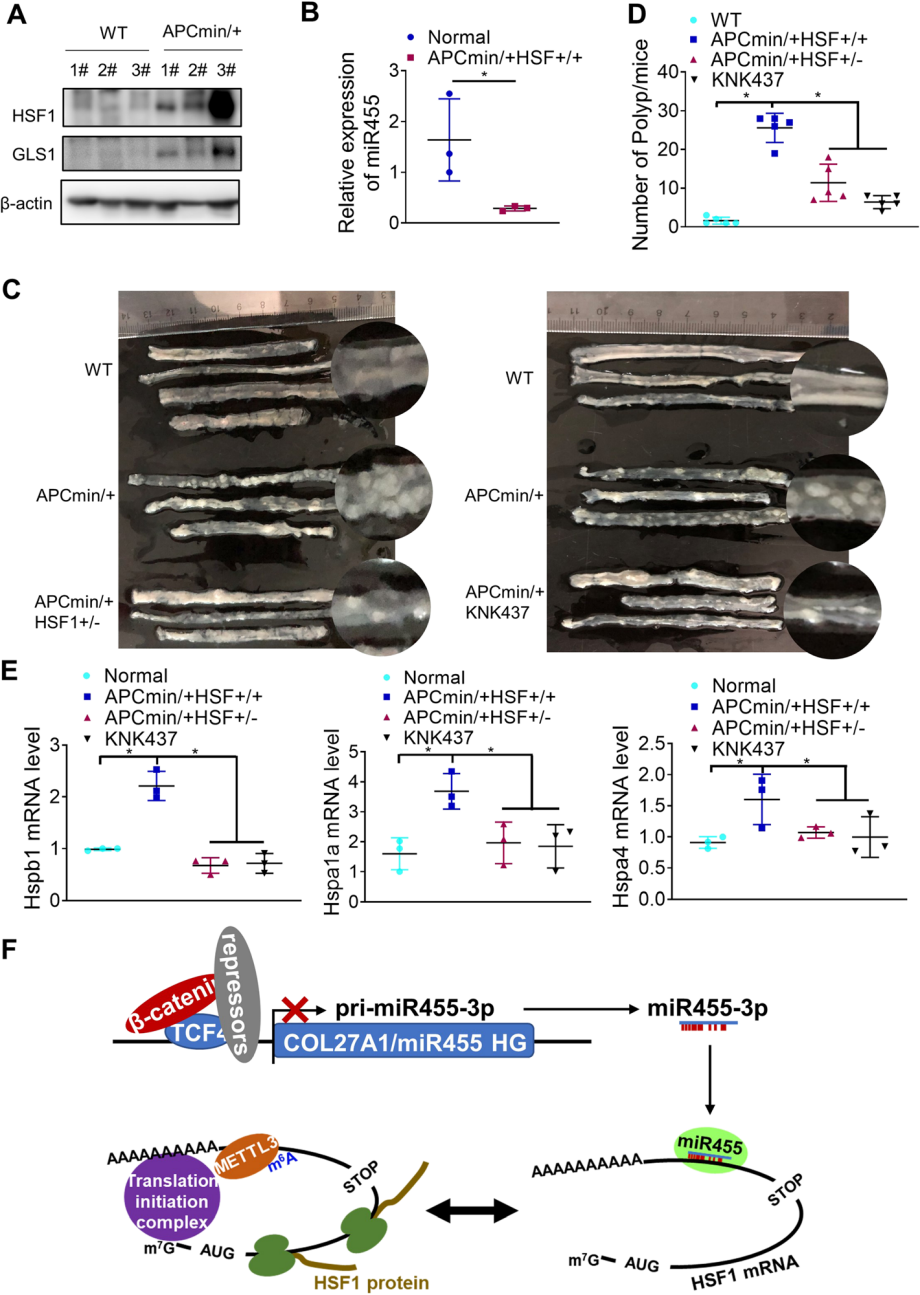
Both genetic and chemical inhibition of HSF1 attenuate colorectal carcinogenesis driven by active Wnt signaling. **a** Expression of HSF1 protein in bowel tissue of APCmin/+ mice was analyzed by western blotting. **b** miR455-3p expression in bowel tissue of APCmin/+ mice was analyzed by RT-PCR. **c** and **d** The number of polyps per mouse in APCmin/+ and APCmin/+ mice when HSF1 was genetically knocked out or chemically inhibited mice was counted. **e** The downstream targets of HSF1 in APCmin/+ treated with KNK437 and APCmin/+HSF1+/− mice were analyzed by PT-PCR. Asterisks (*) indicate a *P* < 0.05. **f** Working model: When Wnt/β-catenin was inactivated, the transcription of COL27A1, the host gene of miR455-3p (miR455HG), was increased so that more miR455-3p was generated to occupy HSF1 mRNA 3′-UTR and prevent it from METTL3-mediated m6A modification, thus repressing HSF1 translation. In contrast, upon Wnt/β-catenin activation, miR455-3p generation was repressed so that HSF1 mRNA accessible for METTL3 binding and m6A modification. Eventually HSF1 translation was stimulated to promote colorectal carcinogenesis

In light of these in vitro findings, we further explored the relevance of HSF1 to colorectal carcinogenesis in Apc^min/+^ and Apc^min/+^ HSF1^+/−^ mice, since the interaction of mouse miR455-3p and mouse HSF1 mRNA seems to be well conserved (mouse miR455-3p seed sequence: CAGUCCA; the binding site in mouse HSF1 mRNA 3′-UTR: tggactg). The expression of HSF1 and its downstream target GLS1 were increased, while miR455-3p expression was reduced, in intestine tissues from Apc^min/+^ mice compared with normal C57BL/6 mice (Fig. 6a and b). After fed with high-fat diet for 3 months, these Apc^min/+^ mice developed multiple tumors in the intestine (Fig. 6c). However, both the size and number of tumors were significantly reduced in Apc^min/+^ mice treated with a chemical inhibitor of HSF1, KNK437 and Apc^min/+^ HSF1^+/−^ mice (Fig. 6d), accompanied by the downregulation of HSF1 targets (Fig. 6e). All of these results confirmed that HSF1 is a novel downstream target of WNT/β-catenin signaling important to promote CRC development.

## Discussion

Genetic changes in components of WNT/β-catenin signaling such as deletion of the APC gene and CTNNB1 mutations have been frequently detected in many types of human cancers [29]. All of these mutations will eventually lead to the activation of WNT/β-catenin signaling, mainly displayed as the accumulation of β-catenin in the nucleus to activate TCF/LEFs-driven transcription of so-called WNT responsive target genes including well-known oncogenes such as c-MYC and cyclinD1 [30, 31]. However, alternative functions of activated WNT/β-catenin signaling were largely overlooked because of the overwhelming relevance of these well-known targets. For example, β-catenin played an important role in mitotic spindle orientation and cell migration through regulating cytoskeleton in a transcription-independent manner [32]. In this study, we revealed a new function of WNT/β-catenin signaling in facilitating mRNA m6A modification and stimulating the synthesis of HSF1 protein, which can activate glutaminolysis to promote the pathogenesis of colorectal carcinogenesis [7].

Recently, RNA modifications such as m6A modification were important for fate decision of target mRNAs [12, 33] . However, the regulation of mRNA m6A modification under various circumstances were largely unknown. We reported here that β-catenin can enhance the interaction of HSF1 mRNA with METTL3 to increase its m6A modification. As a result, the translation of HSF1 mRNA was activated and β-catenin expression was correlated with HSF1 expression in various cell lines and primary tissues. Recently, more and more non-classical RNA binding proteins (RBPs) including β-catenin were found to interact with multiple mRNAs [14, 34]. However, we failed to find the direct interaction of β-catenin with METTL3 or HSF1 mRNA (data not shown), thus excluding the direct recruitment of METTL3 by β-catenin. While the mechanism underlying specific interaction of METTL3 to HSF1 mRNA remains to be investigated, we found that miRNAs can affect the accessibility of HSF1 mRNA to METTL3. METTL3 can interact with HSF1 mRNA only when the abundance of miR455-3p was reduced. Once miR455-3p interacts with HSF1 mRNA by base-pairing, the interaction of METTL3 was greatly impaired. It has been reported that miRNA binding sites were remarkably enriched among m6A modification motifs [35]. Furthermore, overexpressing miRNAs significantly increased the association of target mRNAs with METTL3, whereas downregulating miRNA abundance significantly reduced METTL3 binding on mRNAs [35]. Unfortunately, the consequences of increased m6A modification on these mRNAs were not shown. It has been well-recognized that the interaction of miRNAs will lead to the degradation of target mRNAs or suppression of mRNA translation. In contrast, m6A modification can generate distinct consequences including stimulating mRNA translation, promoting or preventing mRNA degradation. Therefore, miRNA-promoted m6A modification must be restricted in particular cells or circumstances. Generally, expression-suppressing miRNAs should repress m6A modifications promoting mRNA translation, just like what we presented here that miR455-3p inhibited m6A modification of HSF1 mRNA to impair its translation efficiency. Certainly, it remains to be investigated how miRNA binding exactly interfere the interaction with METLL3. MiRNA binding results in the formation of miRISC, which may impede METTL3 interaction. On the other hand, m6A modification once formed can alter the structure and conformation of mRNA, thus affecting the base-pairing with miRNAs [36]. Therefore, miRNAs and METTL3 can compete with each other to control the fate decision of mRNAs. Indeed, only wild type miR455-3p but not its mutant with mutations in the seed sequence, interacted with HSF1 mRNA and prevented METTL3 binding. When the increase of miR455-3p was neutralized by miR455-3p inhibitor, β-catenin depletion cannot reduce m6A modification of HSF1 mRNA anymore.

As a result, the abundance of miR455-3p is critical to regulate m6A modification of HSF1 mRNA. Interestingly, we found that β-catenin inhibited the biogenesis of miR455-3p by suppressing the transcription of its host gene COL27A. While β-catenin was well-known to be a transcriptional co-activator, it can also function as a transcriptional repressor to directly repress gene transcription [37, 38]. For example, β-catenin in complex with LEF1 repressed the transcription of Runx2 targets such as osteocalcin 2 [39]. Actually, we found more genes were downregulated after LiCl treatment than genes upregulated (Additional file 2, Fig.S6A). Similar to translational activation, transcriptional repression function of β-catenin depends on its interaction with TCF/LEF1 but recognizes a distinct TCF-binding motif, AGAWAW instead of CTTTGWWS [40]. Conversion of this unique motif to standard TCF-binding sites switched the mode of regulation from β-catenin-mediated repression to activation, indicating the importance of this motif to transcription inactivation. In consistence with the downregulation of COL27A1 expression by β-catenin, multiple such non-canonical TCF4-binding elements were found in its promoter region. When bound to these motifs by its HMG (high mobility group) domain, TCF can activate gene transcription in the absence of β-catenin. Upon the activation of WNT signaling, β-catenin interacted with TCF and repressed gene transcription although the binding of TCF to these motifs was not altered [41]. Other co-activators and co-repressors are likely to be involved in this allosteric regulation and warrants further investigations. Nevertheless, β-catenin/TCF complex could interact with other transcriptional co-repressors such as members of the Groucho family [42]. Alternatively, β-catenin/TCF complex might be able to inhibit the transactivation activity of other transcription factors interacted with the same promoter.

Taken together, β-catenin suppressed the biogenesis of miR455-3p to allow METTL3 binding for m6A modification of HSF1 mRNA, thus stimulating HSF1 mRNA translation. In cancer cells, HSF1 can drive a transcriptional program distinct from heat shock response to support malignant phenotypes [15, 43]. For instance, HSF1 can drive the transcription of PDK3 (Pyruvate Dehydrogenase Kinase 3) to promote glycolysis in chemo-resistant cancer cells [44]. It can also recruit DNMT3a to the promoter of miR137HG and repress the biogenesis of GLS1 (Glutaminase 1)-targeting miR137, thus upregulating GLS1 expression to activate mTOR and promote cancer development [7]. Currently, there are several ongoing clinical trials to evaluate the potential of targeting HSF1 for cancer treatment. Although WNT signaling has been well-known to be important for the development of CRC and many other cancers, no targeting drugs have been developed for clinical application. This study revealed HSF1 as a novel downstream target for β-catenin to promote cancer development, indicating that HSF1-targeting molecules could be tried for the intervention of human cancer driven by activated WNT/β-catenin signaling.

## Conclusions

In summary, β-catenin suppressed the biogenesis of HSF1-targeting miR455-3p to favor METTL3 binding and m6A modification of HSF1 mRNA, thus promoting HSF1 translation. HSF1 expression was upregulated in murine tumors driven by activated WNT/β-catenin signaling and human CRC tissues. Genetic and chemical inhibition of HSF1 significantly attenuated the pathogenesis of CRC in mice. Targeting HSF1 is a potential strategy for the management of human cancers associated with activated WNT/β-catenin signaling.

## Supplementary information

**Additional file 1: Table S1.** RNA oligonucleotide sequences. **Table S2.** Primer sequences.

**Additional file 2: Supplemnetary Figures 1-6. Fig. S1.** WNT/β-catenin signaling activates HSF1.** Fig. S2.** β-catenin has no effects on the RNA and protein half-life of HSF1. **Fig. S3.** The effect of microRNAs on HSF1 expression in CRC. **Fig. S4.** m6A modification of HSF1 mRNA. **Fig. S5.** The effects of miR455-3p and m6A modification on HSF1. **Fig. S6.** Volcano plot displays differentially regulated genes upon LiCl treatment.

**Additional file 3.** The list of genes containing HSE in their promoters.

## Data Availability

The authors declare that all the other data supporting the findings of this study are available within the article and its additional files and from the corresponding author upon reasonable request. The data for high-throughput sequencing are deposited at GEO under the accession number GSE151119.

## Abbreviations

CRC: Colorectal cancer
HSF1: Heat shock factor 1
HSE: Heat shock response elements
ChIP: Chromatin immunoprecipitation
LiCl: Lithium chloride
MEF: Mouse embryonic fibroblast
UTR: Untranslated region
Acetyl-CoA: Acetyl coenzyme A
TCA: Tricarboxylic acid
GLS1: Glutaminase 1
miRISC: miRNA-induced silencing complex
GSK3β: Glycogen synthase kinase3β
TCF/LEF: T cell-specific factor/lymphoid enhancer binding factor
m6A: N6-methyladenosine
IHC: Immunohistochemical
meRIP: m6A RNA immunoprecipitation
PCR: Polymerase chain reaction

## References

[1] Siegel RL, Miller KD, Jemal A (2019). Cancer statistics, 2019. CA Cancer J Clin.

[2] Rosenbluh J, Nijhawan D, Cox AG, Li X, Neal JT, Schafer EJ, Zack TI, Wang X, Tsherniak A, Schinzel AC (2012). beta-catenin-driven cancers require a YAP1 transcriptional complex for survival and tumorigenesis. Cell.

[3] Morris JP, Yashinskie JJ, Koche R, Chandwani R, Tian S, Chen CC, Baslan T, Marinkovic ZS, Sanchez-Rivera FJ, Leach SD (2019). alpha-Ketoglutarate links p53 to cell fate during tumour suppression. Nature.

[4] Wang Y, Xia Y, Lu Z (2018). Metabolic features of cancer cells. Cancer Commun (Lond).

[5] Hay N (2016). Reprogramming glucose metabolism in cancer: can it be exploited for cancer therapy?. Nat Rev Cancer.

[6] Altman BJ, Stine ZE, Dang CV (2016). From Krebs to clinic: glutamine metabolism to cancer therapy. Nat Rev Cancer.

[7] Li J, Song P, Jiang T, Dai D, Wang H, Sun J, Zhu L, Xu W, Feng L, Shin VY (2018). Heat shock factor 1 epigenetically stimulates Glutaminase-1-dependent mTOR activation to promote colorectal carcinogenesis. Mol Ther.

[8] Eulalio A, Huntzinger E, Izaurralde E (2008). Getting to the root of miRNA-mediated gene silencing. Cell.

[9] Choe J, Lin S, Zhang W, Liu Q, Wang L, Ramirez-Moya J, Du P, Kim W, Tang S, Sliz P (2018). mRNA circularization by METTL3-eIF3h enhances translation and promotes oncogenesis. Nature.

[10] Jonas S, Izaurralde E (2015). Towards a molecular understanding of microRNA-mediated gene silencing. Nat Rev Genet.

[11] Mathonnet G, Fabian MR, Svitkin YV, Parsyan A, Huck L, Murata T, Biffo S, Merrick WC, Darzynkiewicz E, Pillai RS (2007). MicroRNA inhibition of translation initiation in vitro by targeting the cap-binding complex eIF4F. Science.

[12] Wang X, Zhao BS, Roundtree IA, Lu Z, Han D, Ma H, Weng X, Chen K, Shi H, He C (2015). N(6)-methyladenosine modulates messenger RNA translation efficiency. Cell.

[13] Chasse H, Boulben S, Costache V, Cormier P, Morales J (2017). Analysis of translation using polysome profiling. Nucleic Acids Res.

[14] Zhu L, Zhu Y, Han S, Chen M, Song P, Dai D, Xu W, Jiang T, Feng L, Shin VY (2019). Impaired autophagic degradation of lncRNA ARHGAP5-AS1 promotes chemoresistance in gastric cancer. Cell Death Dis.

[15] Mendillo ML, Santagata S, Koeva M, Bell GW, Hu R, Tamimi RM, Fraenkel E, Ince TA, Whitesell L, Lindquist S (2012). HSF1 drives a transcriptional program distinct from heat shock to support highly malignant human cancers. Cell.

[16] Lamb J, Crawford ED, Peck D, Modell JW, Blat IC, Wrobel MJ, Lerner J, Brunet JP, Subramanian A, Ross KN (2006). The connectivity map: using gene-expression signatures to connect small molecules, genes, and disease. Science.

[17] Barham W, Frump AL, Sherrill TP, Garcia CB, Saito-Diaz K, VanSaun MN, Fingleton B, Gleaves L, Orton D, Capecchi MR (2013). Targeting the Wnt pathway in synovial sarcoma models. Cancer Discov.

[18] Watanabe K, Biesinger J, Salmans ML, Roberts BS, Arthur WT, Cleary M, Andersen B, Xie X, Dai X (2014). Integrative ChIP-seq/microarray analysis identifies a CTNNB1 target signature enriched in intestinal stem cells and colon cancer. PLoS One.

[19] Sato Y, Murakami T, Funatsuki H, Matsuba S, Saruyama H, Tanida M (2001). Heat shock-mediated APX gene expression and protection against chilling injury in rice seedlings. J Exp Bot.

[20] Zamdborg L, Ma P (2009). Discovery of protein-DNA interactions by penalized multivariate regression. Nucleic Acids Res.

[21] Liu J, Xu Y, Stoleru D, Salic A (2012). Imaging protein synthesis in cells and tissues with an alkyne analog of puromycin. Proc Natl Acad Sci U S A.

[22] Lewis BP, Burge CB, Bartel DP (2005). Conserved seed pairing, often flanked by adenosines, indicates that thousands of human genes are microRNA targets. Cell.

[23] Bartosovic M, Molares HC, Gregorova P, Hrossova D, Kudla G, Vanacova S (2017). N6-methyladenosine demethylase FTO targets pre-mRNAs and regulates alternative splicing and 3′-end processing. Nucleic Acids Res.

[24] Harper JE, Miceli SM, Roberts RJ, Manley JL (1990). Sequence specificity of the human mRNA N6-adenosine methylase in vitro. Nucleic Acids Res.

[25] Liu J, Yue Y, Han D, Wang X, Fu Y, Zhang L, Jia G, Yu M, Lu Z, Deng X (2014). A METTL3-METTL14 complex mediates mammalian nuclear RNA N6-adenosine methylation. Nat Chem Biol.

[26] Wang X, Feng J, Xue Y, Guan Z, Zhang D, Liu Z, Gong Z, Wang Q, Huang J, Tang C (2016). Structural basis of N(6)-adenosine methylation by the METTL3-METTL14 complex. Nature.

[27] Du Y, Hou G, Zhang H, Dou J, He J, Guo Y, Li L, Chen R, Wang Y, Deng R (2018). SUMOylation of the m6A-RNA methyltransferase METTL3 modulates its function. Nucleic Acids Res.

[28] Lalevee S, Lapaire O, Buhler M (2014). miR455 is linked to hypoxia signaling and is deregulated in preeclampsia. Cell Death Dis.

[29] Kandoth C, McLellan MD, Vandin F, Ye K, Niu B, Lu C, Xie M, Zhang Q, McMichael JF, Wyczalkowski MA (2013). Mutational landscape and significance across 12 major cancer types. Nature.

[30] He TC, Sparks AB, Rago C, Hermeking H, Zawel L, da Costa LT, Morin PJ, Vogelstein B, Kinzler KW (1998). Identification of c-MYC as a target of the APC pathway. Science.

[31] Tetsu O, McCormick F (1999). Beta-catenin regulates expression of cyclin D1 in colon carcinoma cells. Nature.

[32] Gomez-Orte E, Saenz-Narciso B, Moreno S, Cabello J (2013). Multiple functions of the noncanonical Wnt pathway. Trends Genet.

[33] Zhou J, Wan J, Shu XE, Mao Y, Liu XM, Yuan X, Zhang X, Hess ME, Bruning JC, Qian SB (2018). N(6)-Methyladenosine guides mRNA alternative translation during integrated stress response. Mol Cell.

[34] Kim I, Kwak H, Lee HK, Hyun S, Jeong S (2012). beta-catenin recognizes a specific RNA motif in the cyclooxygenase-2 mRNA 3′-UTR and interacts with HuR in colon cancer cells. Nucleic Acids Res.

[35] Chen T, Hao YJ, Zhang Y, Li MM, Wang M, Han W, Wu Y, Lv Y, Hao J, Wang L (2015). m(6) a RNA methylation is regulated by microRNAs and promotes reprogramming to pluripotency. Cell Stem Cell.

[36] Roost C, Lynch SR, Batista PJ, Qu K, Chang HY, Kool ET (2015). Structure and thermodynamics of N6-methyladenosine in RNA: a spring-loaded base modification. J Am Chem Soc.

[37] Meng Q, Mongan M, Wang J, Xia Y (2018). Repression of MAP 3K1 expression and JNK activity by canonical Wnt signaling. Dev Biol.

[38] Smartt HJ, Greenhough A, Ordonez-Moran P, Talero E, Cherry CA, Wallam CA, Parry L, Al Kharusi M, Roberts HR, Mariadason JM (2012). beta-catenin represses expression of the tumour suppressor 15-prostaglandin dehydrogenase in the normal intestinal epithelium and colorectal tumour cells. Gut.

[39] Kahler RA, Westendorf JJ (2003). Lymphoid enhancer factor-1 and beta-catenin inhibit Runx2-dependent transcriptional activation of the osteocalcin promoter. J Biol Chem.

[40] Hoverter NP, Waterman ML (2008). A Wnt-fall for gene regulation: repression. Sci Signal.

[41] Zhang CU, Blauwkamp TA, Burby PE, Cadigan KM (2014). Wnt-mediated repression via bipartite DNA recognition by TCF in the Drosophila hematopoietic system. PLoS Genet.

[42] Brantjes H, Roose J, van De Wetering M, Clevers H (2001). All Tcf HMG box transcription factors interact with Groucho-related co-repressors. Nucleic Acids Res.

[43] Yang T, Ren C, Lu C, Qiao P, Han X, Wang L, Wang D, Lv S, Sun Y, Yu Z (2019). Phosphorylation of HSF1 by PIM2 induces PD-L1 expression and promotes tumor growth in breast Cancer. Cancer Res.

[44] Xu J, Shi Q, Xu W, Zhou Q, Shi R, Ma Y, Chen D, Zhu L, Feng L, Cheng AS (2019). Metabolic enzyme PDK3 forms a positive feedback loop with transcription factor HSF1 to drive chemoresistance. Theranostics.

