# Supplementary material for: β-catenin represses miR455-3p to stimulate m6A modification of HSF1 mRNA and promote its translation in colorectal cancer
Source: Mol Cancer. 2020 Aug 24;19:129. doi: 10.1186/s12943-020-01244-z (PMC7446108; doi:10.1186/s12943-020-01244-z)
Supplement: Supplementary file 1 — Additional file 1: Table S1. RNA oligonucleotide sequences. Table S2. Primer sequences. [file 12943_2020_1244_MOESM1_ESM.docx]

**β-catenin represses miR455-3p to stimulate m6A modification of HSF1 mRNA and promote its translation in colorectal cancer**

Ping Song^1^, Lifeng Feng^2^, Jiaqiu Li^1^, Dongjun Dai^1^, Liyuan Zhu^2^, Chaoqun Wang^3^, Jingyi Li^2^, Ling Li^2^, Qiyin Zhou^1^, Rongkai Shi^1^, Xian Wang^1, *^, Hongchuan Jin^2, *^

**Supplementary Information**

**Table S1** RNA oligonucleotide sequences.

**Table S2** Primer sequences.

**Table S1** RNA oligonucleotide sequences.

| siRNA | Sequence |
| --- | --- |
| β-catenin-1# | S: GCAGUUGUAAACUUGAUUATT |
|  | AS: UAAUCAAGUUUACAACUGCTT |
| β-catenin-2# | S: GGACACAGCAGCAAUUUGUTT |
|  | AS: ACAAAUUGCUGCUGUGUCCTT |
| METTL3-1# | S: GCACTTGGATCTACGGAAT |
| METTL3-2# | S: CGACTACAGTAGCTGCCTT |
| NC siRNA/mimic | S: UUCUCCGAACGUGUCACGUTT |
|  | AS: ACGUGACACGUUCGGAGAATT |
| 455mimic | S: GCAGUCCAUGGGCAUAUACAC |
|  | AS: GUAUAUGCCCAUGGACUGCUU |
| 455mimic-mt | S: GUCAGUUCUGGGCAUAUACAC |
|  | AS: GUAUAUGCCCAGAACUGACUU |
| 431mimic | S: UGUCUUGCAGGCCGUCAUGCA |
|  | AS: CAUGACGGCCUGCAAGACAUU |
| 184mimic | S: UGGACGGAGAACUGAUAAGGGU |
|  | AS: CCUUAUCAGUUCUCCGUCCAUU |
| 490mimic | S: CAACCUGGAGGACUCCAUGCUG |
|  | AS: GCAUGGAGUCCUCCAGGUUGUU |
| 214mimic | S: UGCCUGUCUACACUUGCUGUGC |
|  | AS: ACAGCAAGUGUAGACAGGCAUU |
| 375mimic | S: UUUGUUCGUUCGGCUCGCGUGA |
|  | AS: ACGCGAGCCGAACGAACAAAUU |
| Inhibitor NC | S: CAGUACUUUUGUGUAGUACAA |
| 455inhibitor | S: GUGUAUAUGCCCAUGGACUGC |
| 214inhibitor | S: GCACAGCAAGUGUAGACAGGCA |
| Biotin-NC | S: UUUGUACUACACAAAAGUACUG |
| Biotin-hsa-miR455-3p | S: GCAGUCCAUGGGCAUAUACAC |

**Table S2** Primer sequences.

| Primers | Sequences |
| --- | --- |
| HSP90AA1 | F: TTTCTGAGAAGCAGGGCACC |
|  | R: GGTCTTGGGTCTGGGTTTCC |
| HSPA4 | F: GTGCATTGCAGTGTGCCATC |
|  | R: GGGCAAATCCTGAGGAGAGC |
| HSPB1 | F: ACGCGGAAATACACGCTGC |
|  | R: TACTTGGCGGCAGTCTCATC |
| HSPH1 | F: AGGCGATCTGTGTTAGATGCT |
|  | R: ATCCGAGGTTTCTCATCCAG |
| DNAJB4 | F: GGAGGAAGGGTTGAAAGGA |
|  | R: ATGAAAGGTGTACCGGAAGG |
| HSPA6 | F: ACAGGAGCACAGGTAAGGCT |
|  | R: TTCATGAACCATCCTCTCCA |
| HSPH1-ChiP-Promoter | F: CGAGCCTTCTGGAAAGATTC |
|  | R: AGGCAGGTTTGAGCCAAT |
| DNAJB4-ChiP-Promoter | F: TTAATCGTGGAGGAGGAGAAA |
|  | R: AGAACAGCAAATGCTACAGGAC |
| HSP90AA1-ChiP-Promoter | F: CACCCGTCACCTTGGCTA |
|  | R: GAAGGTTCGGGAGGCTTCT |
| HSPA4-ChiP-Promoter | F: GGAAGGTGCGGGAAGGTTCG |
|  | R: TTCTTGTCGGATGCTGGA |
| β-catenin | F: GCGCCATTTTAAGCCTCTCG |
|  | R: AAATACCCTCAGGGGAACAGG |
| β-actin-human | F: CACCAACTGGGACGACAT |
|  | R: ACAGCCTGGATAGCAACG |
| DNAJB1 | F: CTGATGTCATTTATCCTGCCA |
|  | R: TTTGAATACGACGGGTATCG |
| HSPA1B | F: AGGGTGTTTCGTTCCCTTTA |
|  | R: CATTCCCAGCCTTTGTAGTG |
| Hspa1a(m) | F: TGGTGCAGTCCGACATGAAG |
|  | R: GCTGAGAGTCGTTGAAGTAGGC |
| Hspb1(m) | F: ATCACTGGCAAGCACGAAGA |
|  | R: GGCCTCGAAAGTAACCGGAA |
| Hspa4(m) | F: CCACAGTCTGATGGCTCCAG |
|  | R: GGTCCACTTGCATCTTCTCCT |
| β-actin(m) | F: TAGGCGGACTGTTACTGAGC |
|  | R: TGCTCCAACCAACTGCTGTC |
| HSF1 | F: GACCAAGCTGTGGACCCTC |
|  | R: CACTTTCCGGAAGCCATACAT |
| 18S-rRNA | F: GTAACCCGTTGAACCCCATT |
|  | R: CCATCCAATCGGTAGTAGCG |
| miR455 | F: GCAGTCCATGGGCATATACAC |
| U6 | F: ACGCAAATTCGTGAAGCGTT |
| miR455-Precursor | F: TCCCTGGCGTGAGGGTATGT |
| miR455-Primary | F: CGCAGCCGCTGTTAGTTAAT |
|  | R: TTAGATGCTAGCACGGCACC |
| mirR-455(m) | F: GCAGUCCACGGGCAUAUACAC |
| U6(m) | F: GCAAATTCGTGAAGCGTTCC |
| HSF1-3’UTR-luciferase | F: GCACTAGTAGGCCCCGGAGGAGCTGG |
|  | R: GCAAGCTTGCCATAGCATAGAGCCTGTCT |
| HSF1-3’UTR-mt -luciferase | F: CTGACTATGAACAACCTGCAGGGCTG  GTTACGGTGTGGCTGTGAAGCCCCAACC |
|  | R: GGTTGGGGCTTCACAGCCACACCGTA  ACCAGCCCTGCAGGTTGTTCATAGTCAG |
| COL27A1 | F: TGGCCTTCGACCTCGACAT |
|  | R: CATGCGGGTTCATCTTCCC |
| COL27A1-ChiP-Promoter | F: CCTCTCTCGGCTTGGGACTT |
| COL27A1-Chip-negative | R: AACTTCGGCAGCTCTGCGTC  F: CGCGTTTCTGGAAATCTCC  R: CATGCTGGCTCTCACTTTTTCT |
