# Supplementary material for: β-catenin represses miR455-3p to stimulate m6A modification of HSF1 mRNA and promote its translation in colorectal cancer
Source: Mol Cancer. 2020 Aug 24;19:129. doi: 10.1186/s12943-020-01244-z (PMC7446108; doi:10.1186/s12943-020-01244-z)
Supplement: Supplementary file 2 — Additional file 2: Supplemnetary Figures 1-6. Fig. S1. WNT/β-catenin signaling activates HSF1. Fig. S2. β-catenin has no effects on the RNA and protein half-life of HSF1. Fig. S3. The effect of microRNAs on HSF1 expression in CRC. Fig. S4. m6A modification of HSF1 mRNA. Fig. S5. The effects of miR455-3p and m6A modification on HSF1. Fig. S6. Volcano plot displays differentially regulated genes upon LiCl treatment. [file 12943_2020_1244_MOESM2_ESM.docx]

**β-catenin represses miR455-3p to stimulate m6A modification of HSF1 mRNA and promote its translation in colorectal cancer**

Ping Song^1^, Lifeng Feng^2^, Jiaqiu Li^1^, Dongjun Dai^1^, Liyuan Zhu^2^, Chaoqun Wang^3^, Jingyi Li^2^, Ling Li^2^, Qiyin Zhou^1^, Rongkai Shi^1^, Xian Wang^1, *^, Hongchuan Jin^2, *^

**Supplementary Information**

**Fig.S1. WNT/β-catenin signaling activates HSF1.**

**Fig.S2. β-catenin has no effects on the RNA and protein half-life of HSF1.**

**Fig.S3. The effect of microRNAs on HSF1 expression in CRC.**

**Fig.S4. m6A modification of HSF1 mRNA.**

**Fig.S5. The effects of miR455-3p and m6A modification on HSF1.**

**Fig.S6. Volcano plot displays differentially regulated genes upon LiCl treatment.**


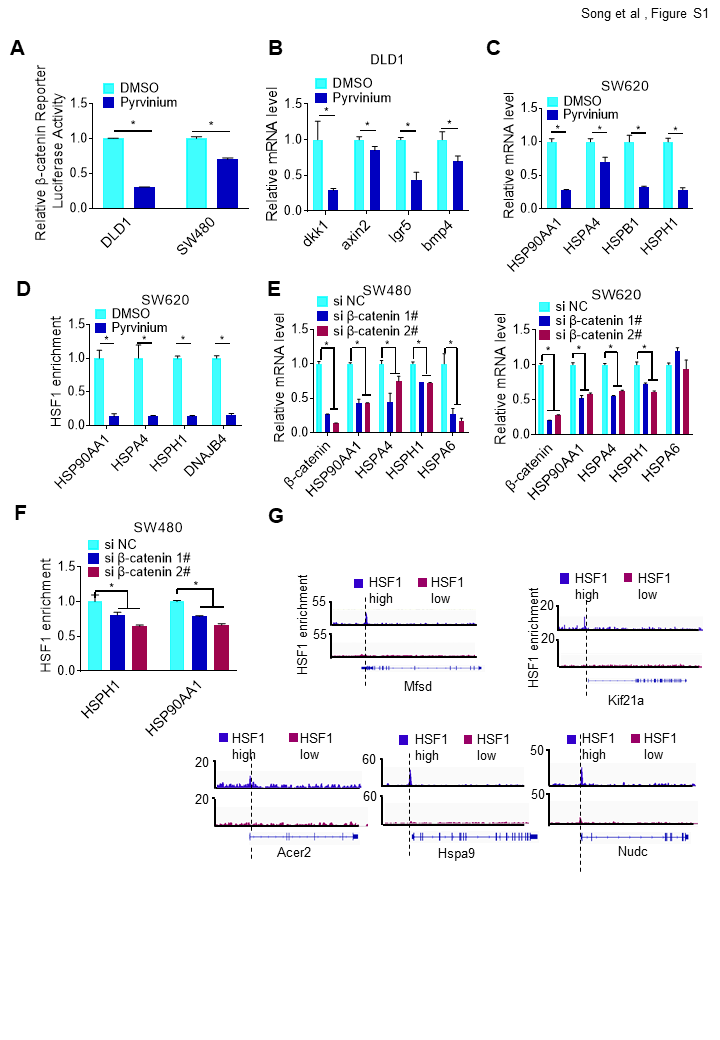
**Fig.S1. WNT/β-catenin signaling activates HSF1.** (**A**) Effects of pyrvinium β-catenin-dependent promoter activity were determined by luciferase activity assay. (**B**) The effect of pyrvinium on the expression of WNT/β-catenin targets were analyzed by RT-PCR. (**C**)The effect of pyrvinium on HSF1 targets were analyzed by RT-PCR. (**D**) Binding of HSF1 to the promoters of HSF1 targets in CRC cells treated with or without pyrvinium was determined by ChIP. (**E**) The mRNA levels of HSF1 targets in CRC cells before and after β-catenin knockdown were analyzed by RT-PCR. (**F**) Binding of HSF1 to its target promoters in CRC cells before and after β-catenin knockdown were analyzed by ChIP. (**G**) Representative HSF1 ChIP-seq tracks (GEO: GSE57398) for 368 genes containing an HSE are shown. *, p<0.05.


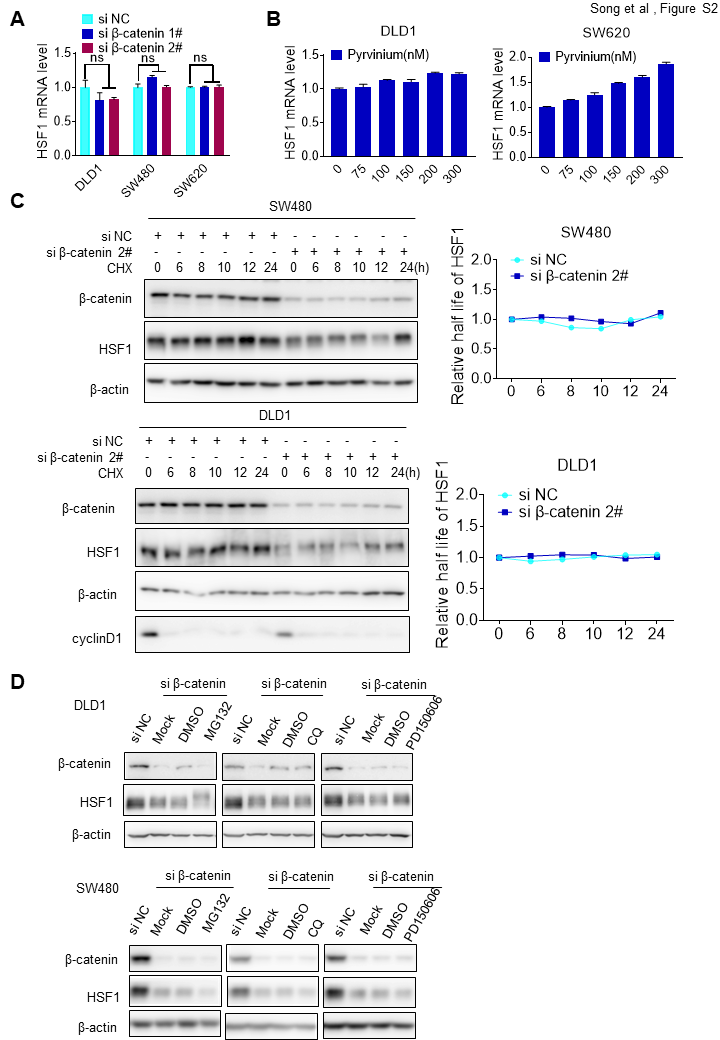


**Fig.S2. β-catenin** **has no effects on the RNA and protein half-life of HSF1.** (**A**) HSF1 mRNA level in CRC cells before and after β-catenin knockdown was analyzed by RT-PCR. (**B**) The effect of pyrvinium on HSF1 mRNA expression was explored by RT-PCR. (**C**)The effect of β-catenin on the half-life of HSF1 protein in CRC cells pretreated with cycloheximide (50μg/mL) was determined by immunoblot analysis. (**D**) The effects of MG132, chloroquine (CQ) and calpin inhibitor PD150606 on β-catenin knockdown-induced HSF1 downregulation were analyzed by western blotting.

**
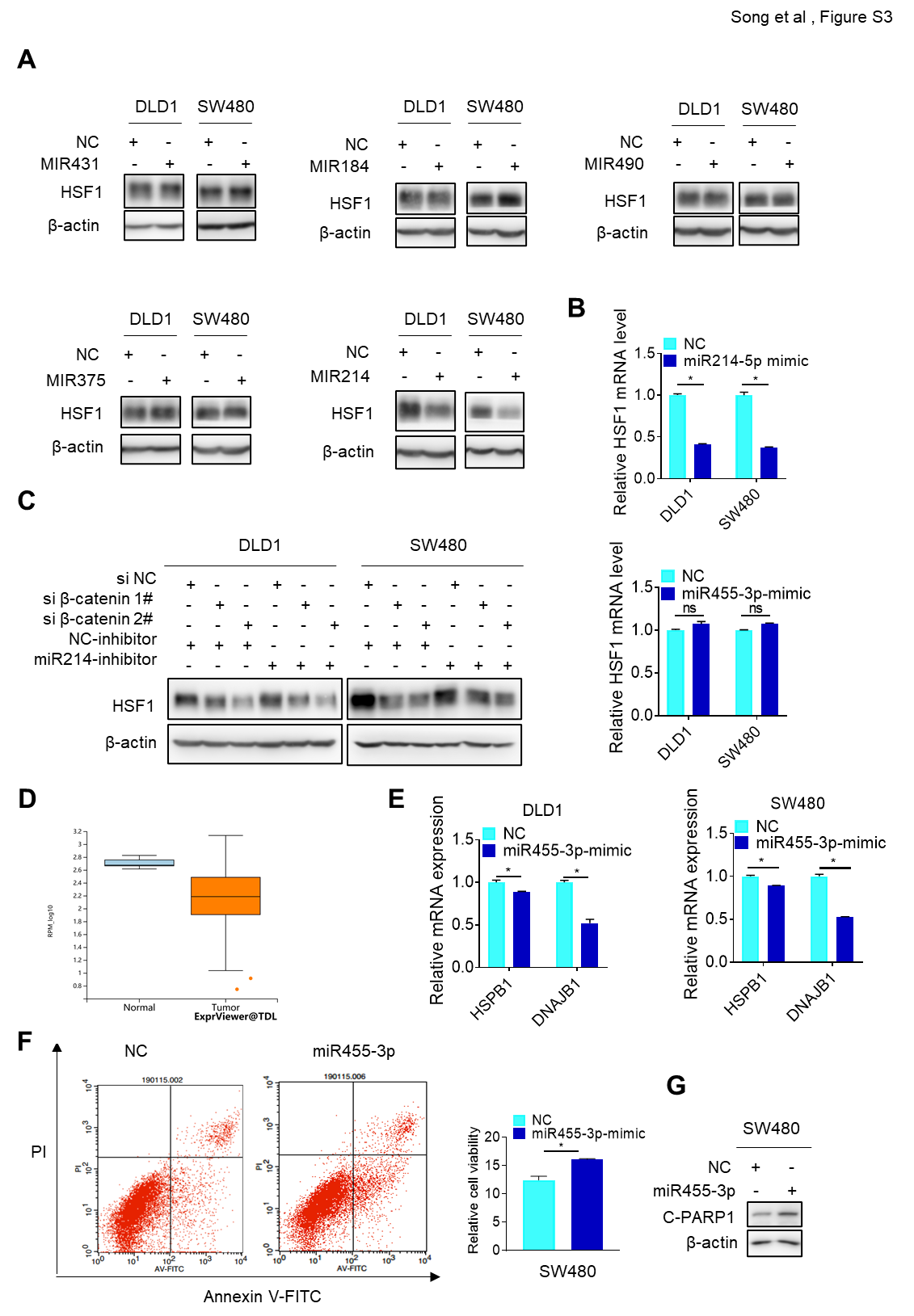
**

**Fig.S3. The effect of microRNAs on HSF1 expression in CRC.** (**A**) The effects of miR431, miR184, miR490, miR375, and miR214 on HSF1 protein expression were analyzed by western blotting. (**B**) The effects of miR214-5p and miR455-3p on the mRNA level of HSF1 were determined by RT-PCR. (**C**) The effect of miR214-5p inhibitor on β-catenin knockdown-induced HSF1 downregulation was determined by western blotting. (D) The expression of miR455-3p in CRC was analyzed on the website (http://mirtv.ibms.sinica.edu.tw/). (E) The effect of miR455-3p on the expression of HSF1 targets were determined by RT-PCR. (**F**) The effect of miR455-3p on apoptosis of SW480 was analyzed using flow cytometry after PI and annexin V- FITC staining. (**G**) Apoptosis of SW480 cells transfected with or without miR455-3p mimics was determined by western blotting.

**
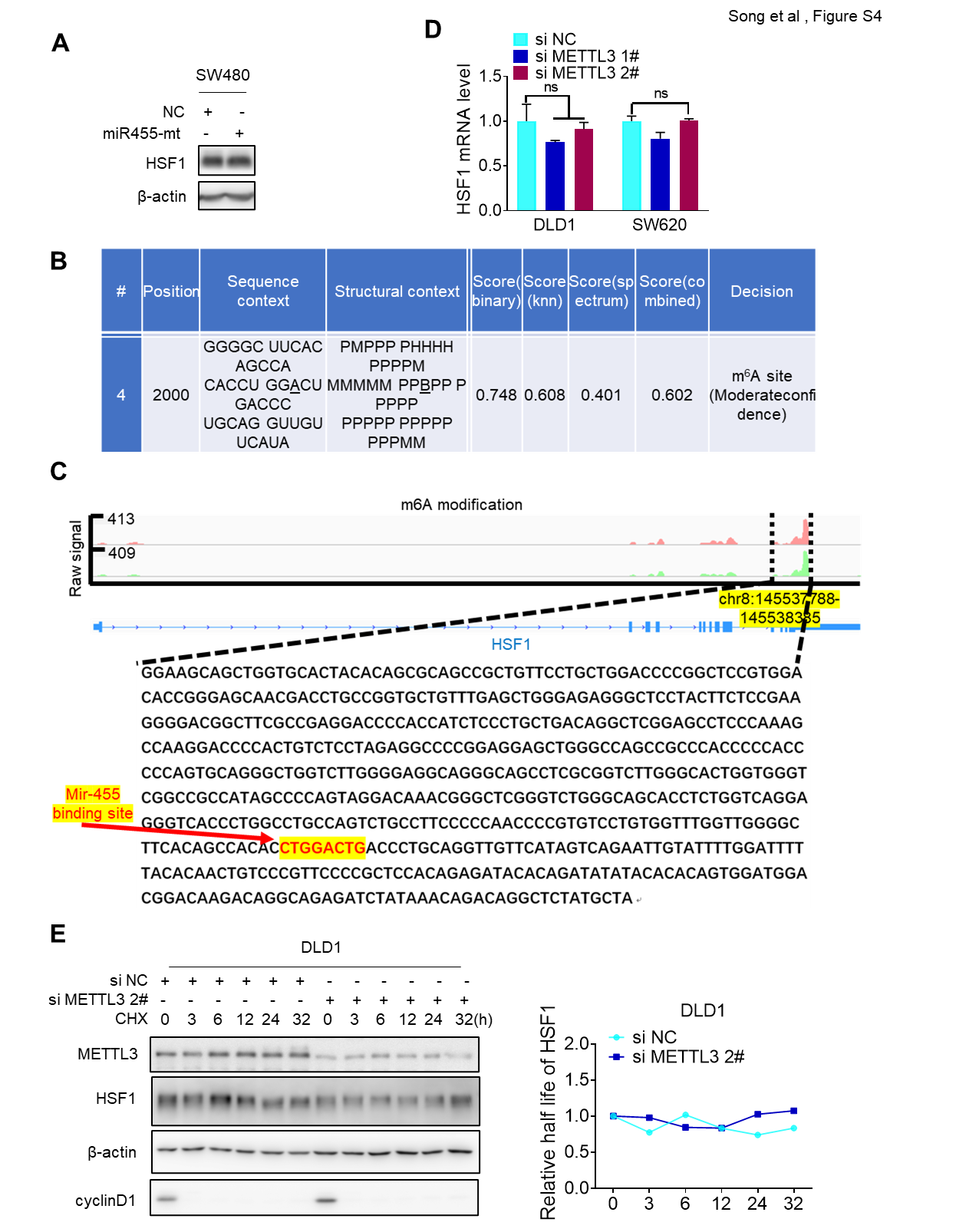
**

**Fig.S4. m6A modification of HSF1 mRNA.** (**A**) HSF1 protein level in SW480 cells with or without mutant miR455-3p transfection was detected by immunoblot analysis. (**B**) Bioinformatic prediction of m6A modification on 3’UTR of HSF1 mRNA. (**C**) The 3’UTR of HSF1 was analyzed by MeRIP seq. (**D**) The effect of METTL3 on the mRNA of HSF1 was analyzed by RT-PCR. (**E**) The effect of METTL3 on the half-life of HSF1 protein in DLD1 cells pretreated with cycloheximide (50μg/mL) were determined by immunoblot analysis.


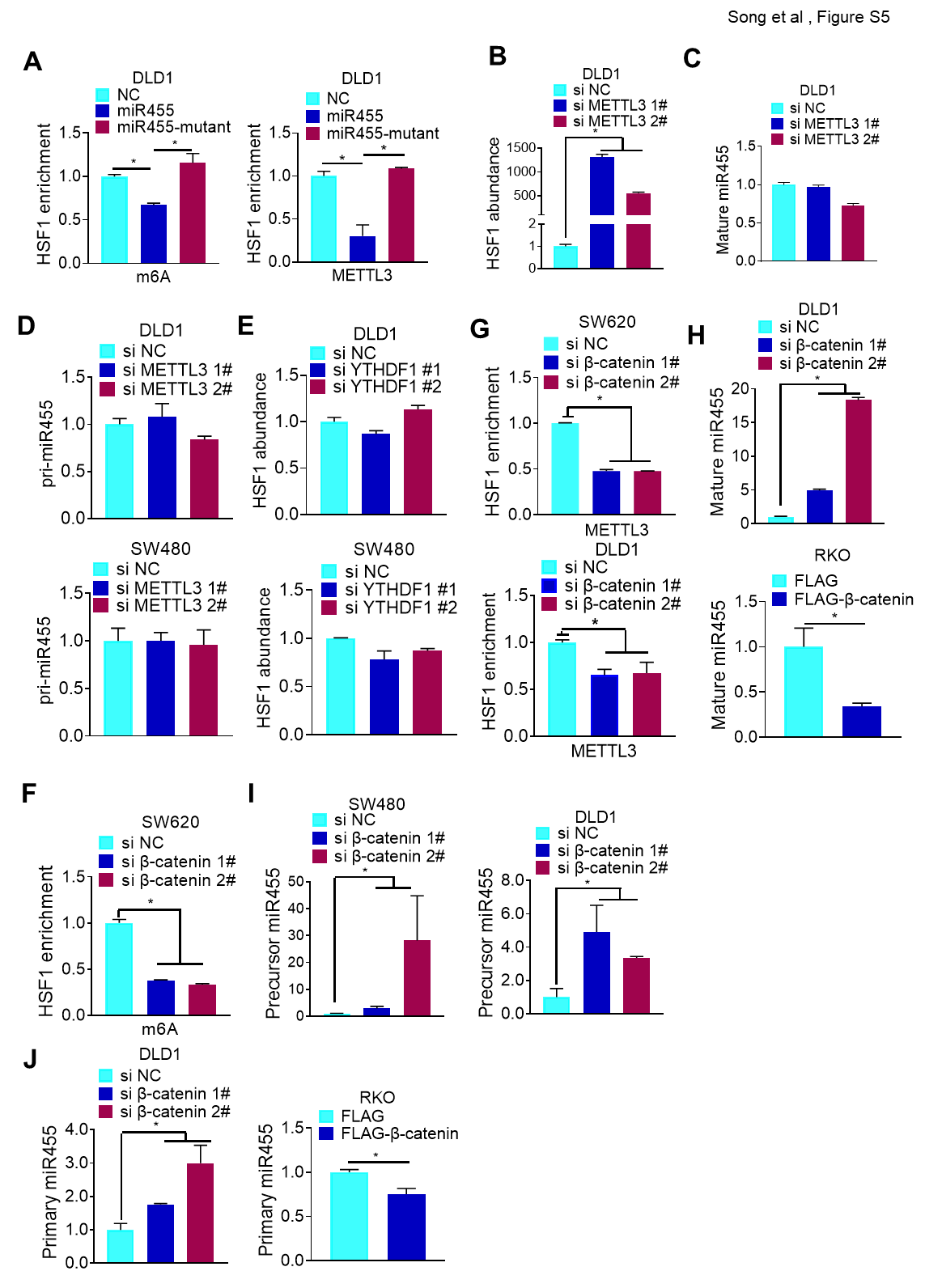


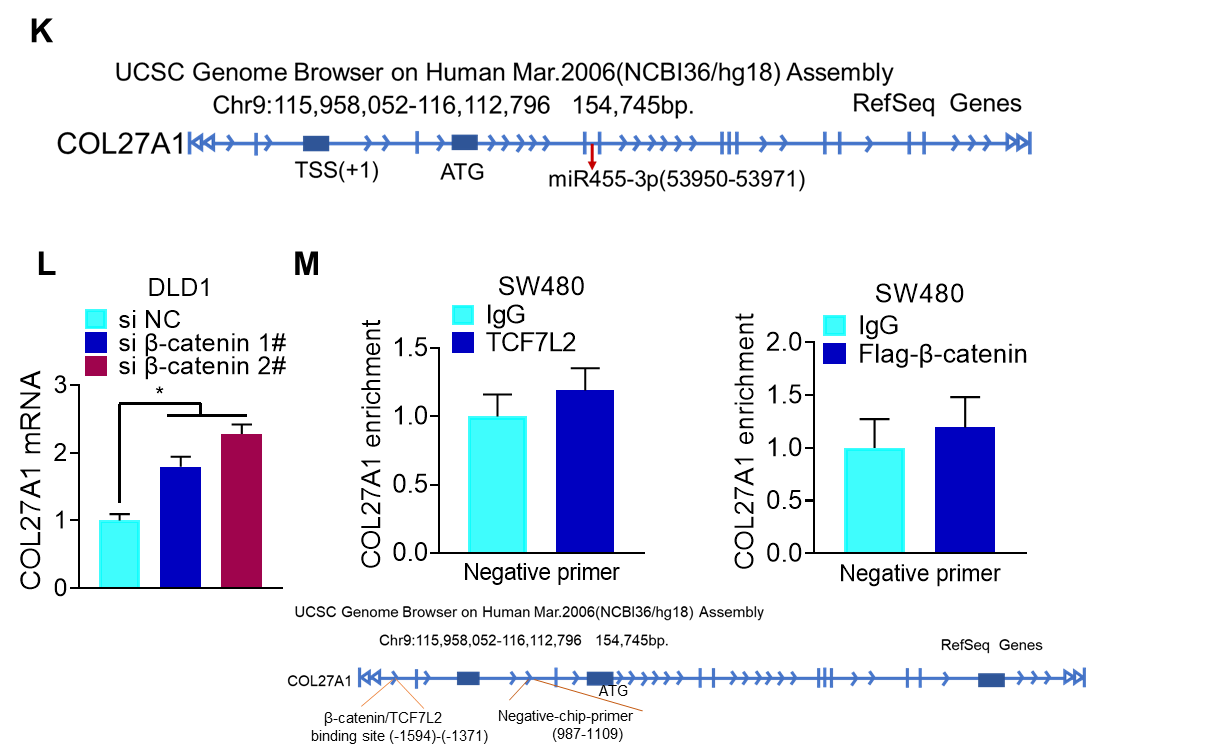


**Fig.S5. The effects of miR455-3p and m6A modification on HSF1.** (**A**) m6A modification and METTL3 binding of HSF1 mRNA in CRC cells transfected with WT or mutant of miR455-3p were analyzed by RIP. (**B**) The interaction between biotin-miR455-3p and HSF1 mRNA with or without METTL3 depletion was analyzed by RT-PCR. (**C**) and (**D**) The levels of mature (C) and primary (D) miR455-3p in CRC cells with or without METTL3 knockdown were determined by RT-PCR. (**E**) The effect of YTHFD1 knockdown on interaction of miR455-3p and HSF1 mRNA was analyzed by biotin pull down. (**F**) and (**G**) The m6A modification of HSF1 (F) and METTL3 interaction of HSF1 mRNA (G) in CRC cells before and after β-catenin knockdown were analyzed by RIP. (**H**) The effects of β-catenin depletion and overexpression on the level of mature miR455-3p were analyzed by RT-PCR. (**I**) The effect of β-catenin depletion on precursor miR455-3p level was analyzed by RT-PCR. (**J**) The effects of β-catenin depletion and overexpression on the level of primary miR455-3p were analyzed by RT-PCR. (**K**) The positional relationship between COL27A1 and miR455-3p was shown in UCSC. (**L**) The effect of β-catenin depletion on mRNA level of COL27A1 was analyzed by RT-PCR. (**M**) The negative-chip-primer was designed away from the β-catenin/TCF7L2 binding site more than 1000bp. *, p<0.05.

**
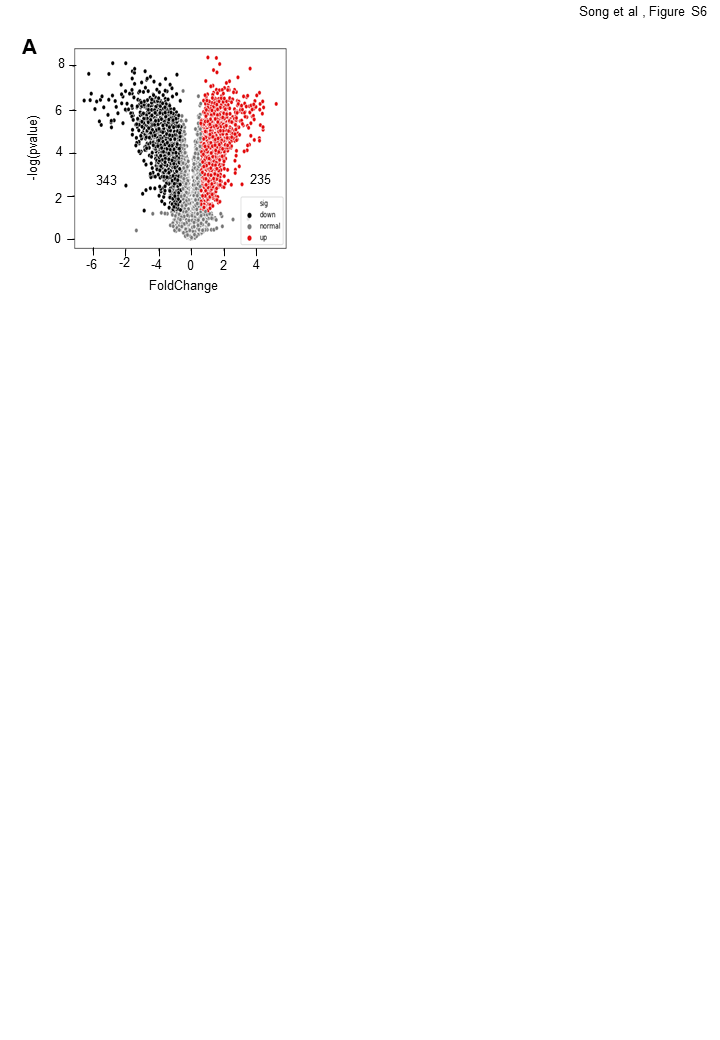
 Fig.S6. Volcano plot displays differentially regulated genes upon LiCl treatment.** Black dots indicate significantly downregulated genes based on adjusted P-value and log-fold change (logFC) (p<0.05, log_2_FC<-2) while red dots represent upregulated genes (p<0.05, log_2_FC>2).
